# Targeting integrin αvβ6 with gallium-68 tris (hydroxypyridinone) based PET probes†

**DOI:** 10.1039/d2dt00980c

**Published:** 2022-08-11

**Authors:** Giuseppe Floresta, Siham Memdouh, Truc Pham, Michelle T. Ma, Philip J. Blower, Robert C. Hider, Vincenzo Abbate, Agostino Cilibrizzi

**Affiliations:** King's College London, Institute of Pharmaceutical Science, Franklin Wilkins Building London SE1 9NH UK agostino.cilibrizzi@kcl.ac.uk; Department of Drug and Health Sciences, University of Catania Catania Italy; King's College London, Division of Imaging Sciences and Biomedical Engineering, Fourth Floor Lambeth Wing, St Thomas’ Hospital London SE1 7EH UK; Centre for Therapeutic Innovation, University of Bath Bath UK

## Abstract

Expression of the cellular transmembrane receptor αvβ6 integrin is mostly restricted to malignant epithelial cells in a wide variety of carcinomas, including pancreatic and others derived from epithelial tissues. Thus, this protein is considered an attractive target for tumour imaging and therapy. Two different ^68^Ga hexadentate tris (3,4-hydroxypyridinone) (THP) chelators were produced in this study and coupled to the αvβ6 integrin–selective peptide *cyclo*(FRGDLAFp(NMe)K) *via* NHS chemistry. Radiolabelling experiments confirmed a high radiochemical yield of the two PET probes. In addition, cellular binding studies showed high binding affinities in the nanomolar range. The two integrin αvβ6-peptide-THP synthesized and radiolabeled in this study will facilitate *in vivo* monitoring of transmembrane receptor αvβ6 integrin by using the advantage of THP chemistry for rapid, efficient and stable gallium chelation.

## Abstract 2

Two ^68^Ga-radiolabeled integrin αvβ6-peptide-THPs to enable *in vivo* monitoring of the transmembrane receptor αvβ6 integrin, by taking advantage of THP chemistry for rapid, efficient and stable gallium chelation.

## Graphical abstract

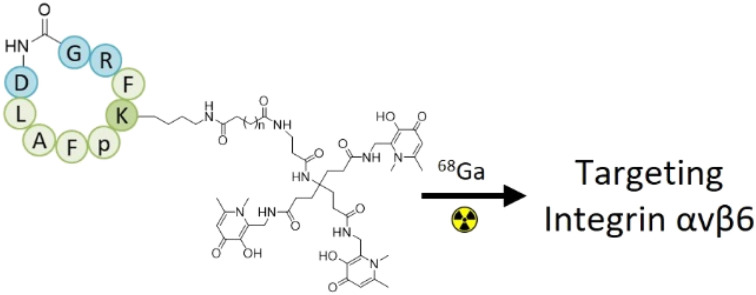

## Introduction

Gamma- and positron-emitting radiometals have potential applications in scintigraphy and positron emission tomography, respectively. To use these radiometals effectively for medical diagnosis, the free radioactive ion must be sequestered from an aqueous solution using a chelating agent and then targeted to the tissue of interest with an address system (*e.g.*, antibodies, nanoparticles or small peptides). When the targeting module, engineered with a chelator, is injected into a patient, it should carry the radioactive isotope without radiometal loss and supply an *in vivo* site-specific radioactive source for imaging or therapy purposes. Today it is feasible to select the specific nuclear properties that are required for a particular therapeutic or diagnostic application. ^68^Ga, ^64^Cu, ^86^Y, ^89^Zr, and ^44^Sc are some examples of radiometals that are currently used clinically for positron emission tomography (PET) imaging.^1^ Interestingly, some radiometals used in PET imaging have multiple radioactive isotopes that are in principle useful for both diagnostic and therapeutic purposes (*e.g.*, ^67/68^Ga, ^60/61/62/64^Cu, ^44/47^Sc, ^86/90^Y). As isotopes of a given element have identical chemical properties, the same radiopharmaceutical ligand can be labelled with different radioisotopes leading to either therapeutic or diagnostic properties.

**Fig. 1:**
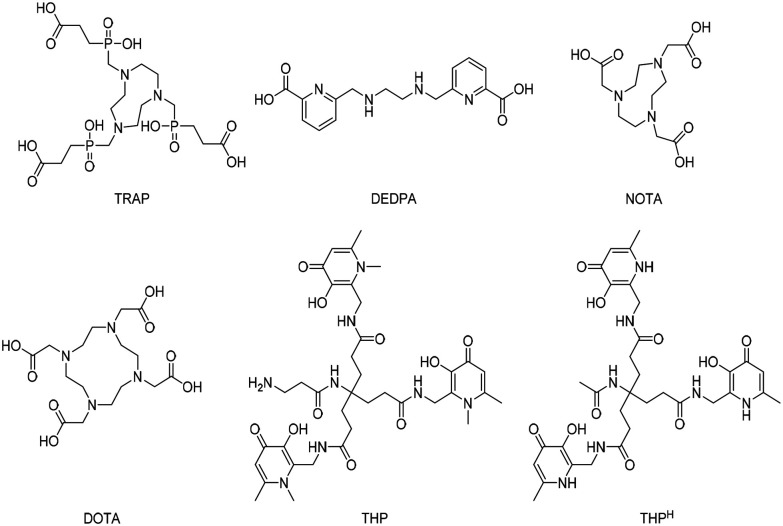
Structures of recently emerged chelators for ^68^Ga.

With the growth of ^18^F and ^11^C based PET imaging tracers, more complex and costly infrastructures have been built to support PET centres within hospitals.^2^ because a kit-based administration is not possible with such ^18^F and ^11^C tracers, requiring the use of on-site cyclotrons and elaborated synthetic chemistry procedures for radiolabelling. In contrast, modern ^68^Ga generators are compatible with good manufacturing practice (GMP) and have the potential to be used in kit-based radiotracers if a simple chelation step can be achieved.^3^ Despite the multitude of efforts on this front, a one-step process for gallium radiolabelling, with the same simplicity as that of the long-established technetium labelling procedures (*i.e.* direct addition of generator eluate to a kit vial), has been elusive to date. Only recently, it was demonstrated that tris (hydroxypyridinone) (THP) based molecules have wide potential in this regard.^4–6^ The majority of adapted ^68^Ga chelators (Fig. 1) do not satisfy the optimal criteria needed for the development of simple one-step kit-based radiotracers. For instance, DOTA is able to bind gallium with extraordinary stability, but it has very slow complexing kinetics that require heating and low pH (in the range of 3.5–5.0) conditions, resulting in low radiochemical yields (<95%) and necessitating a purification step.^7^ TRAP, DEDPA and NOTA (Fig. 1) are other promising candidates, however, in similar fashion to DOTA, gallium chelation requires acidic (or, sometimes, mildly acidic) conditions and the resulting complexes are in some cases susceptible to competition from contaminating trace metals.^8–10^ Although GMP-compliant kit formulations are currently under development also with these chelators, it would appear that the above mentioned disadvantages would prevent the setup of robust simple one-step kit-based protocols. In contrast, THP based molecules possess all the prerequisites to enable one-step kit-based radiolabelling procedures,^6,11^ where the tracers are radiolabelled simply by addition of ^68^Ga generator eluate to a ‘cold’ chelator in the vial (*i.e.* “kit”), in a way which is compatible with GMP, therefore facilitating radio-imaging operations.^3,4^ In this regard, chelators containing the THP scaffold can rapidly chelate ^68^Ga at room temperature and neutral pH with a high yield, not requiring any further purification step for the complexation product. Molecules bearing the THP moiety have been evaluated against a range of commonly employed ligands for ^68^Ga, showing superior properties of radiolabelling under mild conditions.^12–15^ Moreover, in similar fashion to radiopharmaceuticals based on macrocyclic ligands (*e.g.* DOTA), also peptide-THP-based PET tracers have been tested in clinical trials with promising results.^5^

By exploiting the *N*-hydroxysuccinimide (NHS) ester chemistry to generate an easy-to-use kit-based NHS-THP for ^68^Ga-labelling and PET imaging, we recently reported an NHS-functionalized THP-derivative that was successfully conjugated to an anti-programmed death-ligand 1 (PD-L1) camelid single-domain antibody (sdAb) and a glucagon-like peptide-1 (GLP-1) targeting peptide.^16^ In the present study, the NHS-THP chemistry was further explored by developing a new analogue (*i.e.* with a longer linker). The THP-based molecules were then conjugated to an αvβ6 integrin targeted cyclic peptide with the aim of identifying new ^68^Ga-labelled radiopharmaceuticals useful to image tumours *via* αvβ6 integrin.

In this regard, several carcinomas show enhanced αvβ6 integrin expression, such as pancreatic (100% expression),^17^ breast (100% expression)^18^ and oral squamous cell (100% expression) carcinomas,^19–21^ as well as those of the upper aerodigestive tract.^20^ In the context of pancreatic cancer, αvβ6 integrin has been found overexpressed by the malignant cells in almost 90% of all pancreatic ductal adenocarcinomas (PDAC).^22,23^ On this basis, αvβ6 integrin was reasoned to be a promising biomarker for PDAC, which might consequently improve diagnostic and therapeutic approaches toward this tumour.^24,25^ Moreover, αvβ6 integrin levels in healthy tissues are generally low^21^ and expression is confined to epithelial cells.^26^ Interestingly most malignant neoplasms are carcinomas^23^ – *i.e.* tumours of epithelial origin, making the receptor an ideal candidate for the development of a cancer-targeted PET probe.

## Results and discussion

### Design and synthesis of NHS–THP chelators and integrin αvβ6-peptide-THP conjugates

**Fig. 2:**
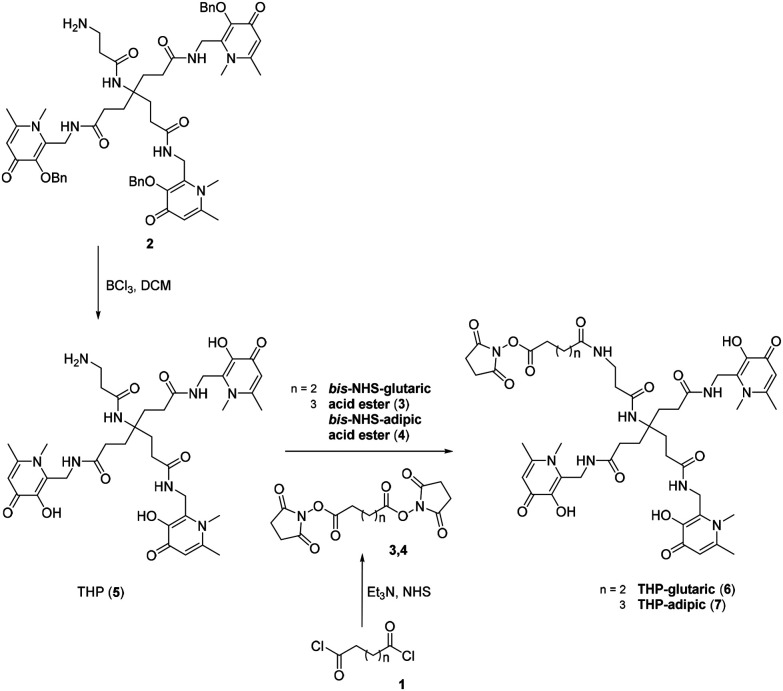
Synthetic scheme for THP-glutaric (6) and THP-adipic (7).

The aim of this study was to produce a kit-based NHS-THP enabling simple and efficient gallium labelling under physiological conditions, by exploiting the recently reported NHS-THP chemistry.^16^ Two THP-derivatives (6, 7) were synthesized. We have previously reported that the 5-carbon atom linker (in 6)^16^ is relatively more stable to hydrolysis of the terminal NHS group than the 4-carbon linker. The stability issue of the 4-carbon linker may be derived from the unique planar structure (with a partially non-saturated C–C bonds, involving sp2 hybridized carbon and nitrogen/planar amide bond) of its molecular skeletons, leading to lower reactivity.^27^ In this work, we have directly compared THP-derivatives with 5- and 6-carbon linkers. We started with the synthesis of the hexadentate tris (3,4-hydroxypyridinone) (2) (Fig. 2),^28^ which was then deprotected with BCl_3_ and conjugated to the two different linkers with either 5- or 6-carbon chains. Two bis-NHS-acid esters were used for the production of the two different linkers – *i.e.* bis-NHS-glutaric acid ester (3) and bis-NHS-adipic acid ester (4), which were synthesized by the reaction of the respective acyl chloride (1) with NHS (Fig. 2), as previously reported.^28^ Treatment of 5 with 10 equiv. of the respective bis-NHS acid ester derivative in DMF produced the two activated esters THP-glutaric (6)^16^ and adipic (7). Both THP-NHS ester derivatives 6–7 presented no stability problems related to the hydrolysis of the terminal NHS group during preparation/purification steps.

**Fig. 3:**
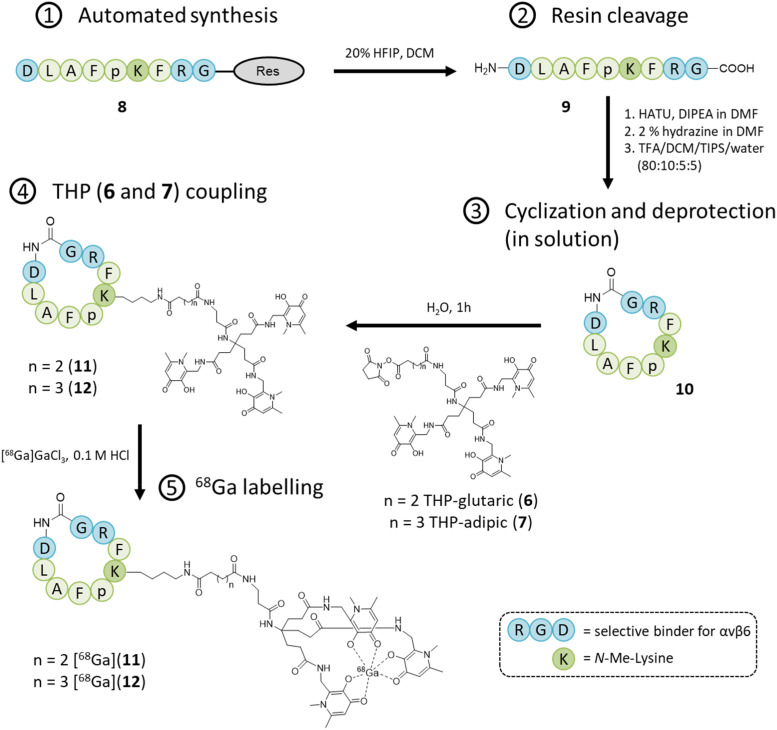
Synthetic scheme for Integrin αvβ6-peptide-THP (11 and 12).

The two molecules 6 and 7 were conjugated to the nonapeptide cyclo-(FRGDLAFp(NMe)K)^29^ (p = D-Pro; (NMe)K = *N*-methyl-Lys) under mild conditions, without the assistance of any activation agent, producing the two conjugates (11 and 12, Fig. 3). Noteworthy, the arginine-glycine-aspartate (RGD) sequence in cyclic peptides is known to selectively bind αv integrins. Moreover, the selected cyclic peptide itself (containing the RGD domain) has been reported as a potent and selective binder for αvβ6, with an IC_50_ of 260 picomolar.^29^ Moreover, the presence of the two non-proteinogenic amino acids, namely *N*-methyl-Lys and D-Pro, might increase the peptide's stability for *in vivo* use.^30,31^ The cyclic peptide 10 (Fig. 3) was assembled *via* standard Fmoc chemistry, using an automated microwave-assisted peptide synthesiser (Fig. 3), which enhanced the efficiency of the overall production of the compound, by substantially reducing the time required to obtain the linear peptide 9 (*i.e. ca.* 7 h, after the coupling of the first amino acid) and increasing its overall purity. The linear peptide (9) was cleaved from the solid support and cyclized in solution with HATU/DIPEA. The cyclic peptide was deprotected (10) and then reacted under mild conditions with the NHS-activated THP derivatives 6 ^16^ and 7 to afford the final compounds, *i.e.* cyclo-(FRGDLAFp(NMe)K)-THPs (11 and 12), which were purified *via* preparative RP-HPLC/UV. The HPLC fractions were further analyzed by analytical RP-HPLC diode array detection (DAD) and the pure fractions were analyzed by mass spectrometry (ESI^+^) (ESI†).

### ^68^Ga radiolabelling of integrin αvβ6-peptide-THP: characterization by radio-HPLC and ITLC

Integrin αvβ6-peptide-THPs 11 and 12 were radiolabelled as described in the Experimental section. Each radiolabelling reaction used ∼30 MBq ^68^Ga and 20 μg of chelator, to give a specific activity of ∼3 GBq μmol^−1^. A purification step was not required after metal complexation/radiolabelling. This confirms the advantage of using THP over other chelators, ensuring to obtain near quantitative radiochemical yields, which can be achieved at low concentrations of chelator, obviating the need to remove unreacted ^68^Ga. Instant thin layer chromatography (ITLC) analysis and radio-HPLC analysis were conducted to evaluate the chelating capability of the two newly synthesized Integrin αvβ6-targeted peptide-THP conjugates (11 and 12) for ^68^Ga. Only 20 μg of 11 or 12 were used for both the radio-HPLC and ITLC analyses (see Experiment section for details). Compound 12 was analyzed by both methods and once its chelating properties had been verified, compound 11 was analyzed only by radio-HPLC. Two different mobile phases (acetate and citrate) were used in the ITLC analysis (Fig. S1†). The citrate method was unable to identify the chelating product, as under these conditions the ^68^Ga itself has a retention factor close to 0 both in the presence and absence of the chelating molecule 12. The acetate method, however, could clearly separate free ^68^Ga from the radiolabelled peptide under the conditions of ITLC analysis. The labelled peptide [^68^Ga]Ga-Integrin αvβ6-peptide-THP (12) has a retention factor of 0.7–0.8 whereas the free ^68^Ga has a retention factor close to 0 (Fig. S1†). Similar results were obtained with the HPLC analyses for both molecules 11 and 12. The results confirmed the formation of [^68^Ga]Ga-Integrin αvβ6-peptide-THP (11) and [^68^Ga]Ga-Integrin αvβ6-peptide-THP (12). Reverse phase HPLC chromatograms showed retention times of 1.9, 11.9 and 13.1 min for unbound ^68^Ga, 11 and 12, respectively. Each analysis gave a single peak in the HPLC radio-chromatograms (Fig. S2 and 3†), and under these conditions, the radiochemical yield for both products was >95% (determined by radio-HPLC).

### Competitive binding assay

**Fig. 4:**
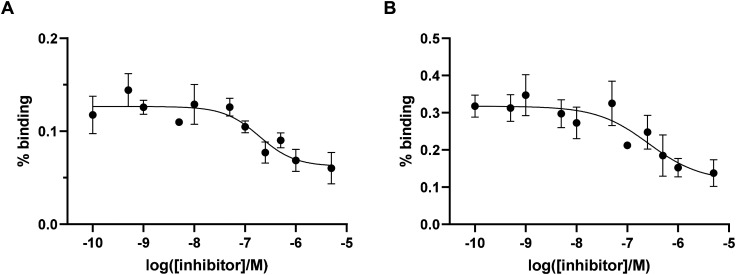
(A) %binding (% added dose) for [^68^Ga]Ga-Integrin αvβ6-peptide-THP (11) in BxPC3 cells (*n* = 4). (B) %binding (% added dose) for [^68^Ga]Ga-Integrin αvβ6-peptide-THP (12) in BxPC3 cells (*n* = 4).

To preliminary estimate the affinity of the probes for integrin αvβ6, IC_50_ values were determined in a competitive binding assay using pancreatic cancer BxPC-3 cells (known to possess a high expression of integrin αvβ6)^32^ and not considering the non-specific binding of the peptide (*i.e.* with no controls to account for non-specific binding). Binding of [^68^Ga]Ga-Integrin αvβ6-peptide-THP (11 and 12) to αvβ6 integrin was determined in the presence of increasing concentrations of unlabelled THP-peptide conjugate (Fig. 4). The IC_50_ values differed between the two ^68^Ga-labelled compounds, with the IC_50_ of the analogue [^68^Ga]Ga-Integrin αvβ6-peptide-THP (12) (260 nM) being higher than that of [^68^Ga]Ga-Integrin αvβ6-peptide-THP (11) (190 nM). Overall, these data confirm that the new agents retain a strong affinity for the αvβ6 integrin target (although lower than the non-conjugated cyclic peptide 10),^29^ demonstrating that functionalisation of the Lys moiety still permits interaction with the protein target, in order to access suitable imaging agents for PET, as well as other visualisation techniques used in diagnostic settings.

## Conclusions and perspective

In this paper we have extended the NHS-THP analogue series and reported a new THP-based bifunctional chelator (7) that could be conjugated under mild conditions to any targeting molecule that contains a primary amine. A cyclic αvβ6 targeted peptide [*i.e. cyclo*(FRGDLAFp(NMe)K)] was conjugated to two NHS-THP analogues *via* NHS chemistry. The resulting bioconjugates (11 and 12) were studied for their gallium chelating capabilities and both resulted in >95% radiochemical yield, proving that the developed NHS chemistry works well and is compatible with THP scaffolds. Moreover, the labelling is efficient even with small quantities (20 μg) of the conjugates, allowing simple preparation of a high effective molar activity product, taking advantages of THP chemistry for gallium efficient chelation. Cellular binding experiments demonstrated nanomolar affinity of the probes on BxPC-3 cells, highlighting their potential for *in vivo* monitoring of the transmembrane receptor αvβ6 integrin. This will enable future preclinical tests *in vivo* with the newly developed probes 11 and 12, in order, to further assess their efficacy as specific PET imaging agents for cancers with high αvβ6 integrin expression, such as pancreatic, ovarian, lung, and gastric carcinomas, as well as invasive head and neck carcinomas. Moreover, the newly developed NHS-THPs confirm that this bioconjugation strategy is a valid tool for the rapid and direct introduction of THP moieties into various targeting modules (*e.g.* peptides, antibodies, anticalins), using either a solid-phase approach such as in this study, or synthetic methodologies in solution, paving the way for the synthesis of various biologically relevant constructs which exploit the advantages of THP chemistry.

## Experimental section

### Synthesis

#### Material

Materials and chemicals were purchased from Fisher scientific, Merck, CEM corporation and were reagent grade or better. Solvents and NMR solvents were purchased from Fisher Scientific, Merck, and VWR. Silica gel for column chromatography was purchased from Merck. Silica gel 60 F254, Merck pre-coated aluminium sheets were employed for thin-layer chromatography (TLC) and spots were visualized under UV light. ^1^H NMR and ^13^C NMR spectra were recorded on A Bruker Avance III HD NanoBay 400 MHz NMR with a 5 mm ^1^H/^13^C/^15^N/^31^P QNP probe equipped with *z*-gradient. Tetramethylsilane (TMS) was used in all NMR experiments as internal standard and chemical shift (*δ*) values are given in ppm. General NMR Analysis Toolbox (GNAT) was used for the analyses of NMR experiments.^30^ Low resolution mass spectra were obtained on a Thermofisher LCQ DECA XP ion trap mass spectrometer or a Waters – Micromass ZQ – Single quadrupole mass spectrometer. Analytical RP-HPLC was carried out on a HP1050 HPLC system equipped with an autosampler, a quaternary pump and a Diode-Array Detector (DAD). The HPLC column was a Zorbax SB C-18 2.1 mm × 10 cm (particle size 5 micron), the employed flow rate was 0.3 mL min^−1^ and the eluents were monitored at wavelengths between 210–280 nm. A linear gradient of mobile phase B (acetonitrile (ACN) containing 0.1% trifluoroacetic acid (TFA)) over mobile phase A (0.1% TFA in water) from 0–90% B in 20 minutes was adopted. Data were collected and analysed using ChemStation software. For further characterization of both final products (compounds 11 and 12), a Thermo Scientific Q-Exactive™ mass spectrometer (hybrid quadrupole orbitrap mass spectrometer) (Thermo Scientific, Bremen, Germany) was used. Direct infusion of both compounds at 1 μg mL^−1^ was carried out in positive ionization mode. The HESI-II source operating conditions using nitrogen were: sheath gas flow rate 70; auxiliary gas flow rate 10; spray voltage 3.75 kV; capillary temperature 320 °C; S-lens RF level 55.0 and auxiliary gas temperature 350 °C. Full MS scan range was *m*/*z* 400–2000 with a resolution of 70 000 FWHM and AGC target of 1 × 10^6^. Collision induced fragmentation (CID) or all ion fragmentation (AIF) experiments with HRMS was performed at *m*/*z* 150–2000 with a resolution of 35 000 FWHM and AGC target of 1 × 10^6^. Xcalibur™ (version 4.1, Thermo Scientific) was used for data analysis.

### Synthesis of THP (5)

Synthesis of compound 5 was conducted as previously reported.^16^ Lyophilized 2 ^33^ (1 mmol) was dissolved in anhydrous dichloromethane (DCM) (1 mL) and cooled to 0 °C. An excess of BCl_3_ (5 mL of a solution 1 M in DCM) was dropwise added and the solution of compound 2 and BCl_3_ was stirred under nitrogen overnight at room temperature. The solution was chilled in an ice bath to 0 °C and methanol (50 mL) was added to quench the excess of BCl_3_. The reaction was left under stirring for additional 1 hour. The solution was then evaporated under reduced pressure, dried under high vacuum for 3 h, dissolved in a minimal amount of methanol (0.5 mL) and precipitated in 0 °C diethyl ether. Following centrifugation, the pellet was washed three times with diethyl ether, dissolved in 50% ACN in water containing 0.1% TFA and freeze-dried for 24 h. The freeze-dried compound 5 was used without further purification. Yield: 99%. ^1^H NMR (400 MHz, DMSO-*d*_6_) *δ* 1.81 (t, *J* = 8.6 Hz, 2H), 2.12 (dd, *J* = 11.3, 5.1 Hz, 2H), 2.66–2.42 (m, 5H), 2.98–2.83 (m, 1H), 3.90 (s, 3H), 4.57 (d, *J* = 5.1 Hz, 2H), 7.33 (d, *J* = 4.0 Hz, 1H), 8.09 (s, 1H), 9.02 (s, 1H). ^13^C NMR (101 MHz, DMSO-*d*_6_) *δ* 21.11, 29.79, 30.51, 32.99, 35.24, 35.65, 39.34, 39.54, 39.75, 39.96, 40.17, 40.38, 40.59, 57.61, 113.23, 140.55, 143.10, 149.07, 159.92, 169.64, 173.89. Calculated ESI^+^ [M + H]^+^: 769.4. Measured ESI^+^ [M + H]^+^: 769.4.

### General procedure for the synthesis of bis-NHS-glutaric/adipic acid ester (3 and 4)

To solution of dry tetrahydrofuran (10 mL) and *N*-hydroxysuccinimide (27.7 mmol), triethylamine (27.7 mmol) was added at 0 °C. glutaryl chloride 3 or adipoyl chloride 4 (12.6 mmol) was added dropwise over 10 min, and the resulting suspension was stirred for 2 h at room temperature. The solvent was then evaporated under reduced pressure. The residue was then dissolved in DCM (100 mL) and washed three times with water (50 mL). The organic extract was dried over MgSO_4_. Then filtration was followed by evaporation of the solvent to yield 3 (90%) and 4 (89%) as white solids, after recrystallization from isopropyl alcohol.^28^

### General procedure for the synthesis of THP-glutaric/adipic acid ester (6 ^16^ and 7)

Compound 5 (1 mmol) was dissolved in 1 mL of dimethylformamide (DMF) and *N*,*N*-diisopropylethylamine (DIPEA) (6 equiv., 6 mmol) was added to the solution. A solution of bis-NHS acid ester derivative (3 or 4) (10 mmol) in DMF (5 mL) was prepared and the solution of compound 5 was added into it dropwise at 0 °C. The reactions were completed within 3 h, as established by direct infusion analysis into the MS and HPLC-UV. The crude of the reaction was dried under vacuum and chromatographed *via* preparative HPLC/UV using H_2_O/0.1% TFA as eluent A and ACN/0.1% TFA as eluent B. The elution program used a linear gradient of 0%–60% of eluent B in 60 min. The detection wavelength was 281 nm and the flow rate was 15 mL min^−1^. THP-glutaric 6; yield: 85%.^16^ THP-adipic 7; yield: 90%; ^1^H NMR (400 MHz, methanol-*d*_4_) *δ* 6.95 (s, 3H), 4.70 (s, 6H), 3.94 (s, 9H), 3.75–3.70 (m, 4H), 3.24–3.22 (m, 4H), 2.58 (s, 9H), 2.31 (m, 6H), 2.23–2.19 (m, 6H), 1.96 (d, *J* = 8.6 Hz, 4H), 1.65–1.62 (m, 4H); calculated ESI^+^ [M + H]^+^: 994.5, [M + 2H]^2+^: 497.7, measured ESI^+^ [M + H]^+^: 994.5, [M + 2H]^2+^: 497.8.

### Synthesis of integrin αvβ6-peptide (8 and 9) and integrin αvβ6-peptide-THP (11 and 12)

The precursor linear sequence was synthesized using standard Fmoc solid-phase peptide synthesis approach at peptide synthesizer (Biotage® Initiator + Alstra™). All the Fmoc-(fluorenylmethyloxycarbonyl)-amino acid residues contained in the sequence were side-chain protected with classical acid-labile protecting groups except from the *N*-methyl-Lys that was protected with the – Dde: Fmoc-Asp(*t*Bu)-OH – Fmoc-Leu-OH – Fmoc-Ala-OH – Fmoc-Phe-OH – Fmoc-D-Pro-OH – Fmoc-NMe-Lys-Dde-OH – Fmoc-Phe-OH – Fmoc-Arg(Pbf)-OH – Fmoc-Gly-OH. 2-Chlorotrityl chloride resin (loading 1.47 mmol g^−1^, 0.2 g) was used as solid support. For the loading of the resin 2 eq. of Fmoc-protected amino acid Fmoc-Gly-OH, 2 equiv. of DIPEA (*N*,*N*-diisopropylethylamine) were dissolved in DCM (2 mL) and the reaction mixture was added to a previously swollen resin in DCM. The reaction mixture was allowed to mix on a tube roller mixer overnight at room temperature. The resin was then washed three times with DCM (20 mL) and DMF (20 mL). For Fmoc-deprotection the resin was treated two times for 15 min with 20% piperidine/DMF (10 mL). A standard protocol was then used for the peptide elongation under microwave irradiation using the peptide synthesizer. In each step the resin was reacted with 1.2 equiv. of Fmoc-protected amino acid, 1.2 equiv. of ‘oxyma’ (ethyl 2-cyano-2-(hydroxyimino)acetate) and 1.2 equiv. of DIC in DMF. Fmoc-deprotections were performed with 20% piperidine/DMF (10 mL). Coupling steps were made in 5 min at 75 °C, deprotections steps were made in 35 min room temperature. At the end of the sequence, the last amino acid (aspartic acid) was Fmoc deprotected and the peptide was cleaved from the resin leaving the side chain protecting groups untouched by addition of hexafluoroisopropanol (HFIP; 20% in DCM, v/v) (3 × 10 min). The crude product (9) was analyzed by RP-HPLC and mass spectrometry (purity >85%) and used in the next step. The linear product was then cyclized in solution (0.001 M concentration)^34^ using HATU (1.5 eq.), DIPEA (1.5 eq.) in DMF. The Dde-protecting group was subsequently cleaved from the lysine side chain with hydrazine (2% in DMF, v/v, 3 × 10 min). The solvent was removed under vacuum and the peptide was dissolved in the following acidic cleavage mixture to deprotect all the remaining side chain protecting groups: trifluoroacetic acid (TFA)/DCM/triisopropylsilane (TIPS)/water (80 : 10 : 5 : 5) for 1.5 h at room temperature. The solution was then concentrated, and the crude product was isolated by precipitation into cold diethyl ether. The precipitate was collected by centrifugation and dried under vacuum. The final cyclic compound (10) was purified *via* preparative HPLC/UV with a Gemini 5 μ C_18_ 110A/AXIA column (100 × 30 mm × 5 micron), using 0.1% TFA in H_2_O as eluent A and 0.1% TFA in ACN as eluent B. The elution program used a linear gradient of 0%–40% of the eluent B in 60 min. The detection wavelength was 281 nm, and the flow rate was 15 mL min^−1^. The isolated fractions were further analyzed by analytical HPLC-DAD, using the same above-mentioned mobile phase of the preparative purification. The elution program used a linear gradient of 0%–95% of eluent B in 20 min using a C_18_ column at a 0.3 mL min^−1^ flow rate. The NHS-THP (6 and 7) were then site-specifically (Lys of 10) coupled under mild conditions using 2 equiv. of THP in water solution by shaking the solution for 1 h without the use of activation agents. Final compounds Integrin αvβ6-peptide-THP (11 and 12) were then purified by preparative HPLC/UV using 0.1% TFA in H_2_O as eluent A and 0.1% TFA in ACN as eluent B. The elution program used a linear gradient of 0%–40% of the eluent B in 60 min. The detection wavelength was 281 nm, and the flow rate was 15 mL min^−1^. The isolated fractions were further analyzed by analytical HPLC-DAD, using the same above-mentioned mobile phase of the preparative purification. The elution program used a linear gradient of 0%–95% of eluent B in 20 min using a C_18_ column at a 0.3 mL min^−1^ flow rate. The pure fractions were then lyophilized and obtained as white solid (yield 60–70%, for the last step after preparative purification). 11: ^1^H NMR (400 MHz, DMSO-*d*_6_) *δ* 8.64–8.56 (m, 2H), 8.49 (t, *J* = 5.3 Hz, 3H), 8.02 (d, *J* = 7.4 Hz, 1H), 7.91–7.85 (m, 1H), 7.77 (t, *J* = 5.3 Hz, 1H), 7.74–7.55 (m, 2H), 7.50 (t, *J* = 5.8 Hz, 1H), 7.40 (d, *J* = 9.0 Hz, 1H), 7.36–7.07 (m, 10H), 6.91 (s, 3H), 4.88–4.76 (m, 2H), 4.59–4.45 (m, 8H), 4.45–4.28 (m, 2H), 4.00–3.92 (m, 1H), 3.78 (s, 9H), 3.72 (d, *J* = 5.3 Hz, 1H), 3.48 (d, *J* = 8.6 Hz, 1H), 3.25 (s, 1H), 3.18 (q, *J* = 6.8 Hz, 2H), 3.09 (p, *J* = 6.6 Hz, 1H), 3.03–2.96 (m, 5H), 2.83–2.79 (m, 1H), 2.74–2.62 (m, 1H), 2.62–2.54 (m, 1H), 2.35–2.33 (m, 1H), 2.21–2.17 (m, 2H), 2.12–1.89 (m, 13H), 1.79 (s, 6H), 1.68–1.63 (m, 2H), 1.58–1.35 (m, 2H), 1.35–1.17 (m, 4H), 1.01 (s, 2H), 0.88–0.83 (m, 5H). Calculated ESI^+^ [M + 2H]^2+^: 956.5, [M + 3H]^3+^: 637.7, [M + 4H]^4+^: 478.8. Measured ESI^+^ [M + 2H]^2+^: 957.4, [M + 3H]^3+^: 638.7, [M + 4H]^4+^: 479.3. 12: ^1^H NMR (400 MHz, DMSO-*d*_6_) *δ* 8.62–8.54 (m, 2H), 8.50 (t, *J* = 5.3 Hz, 3H), 8.01 (d, *J* = 7.4 Hz, 1H), 7.75 (t, *J* = 5.3 Hz, 1H), 7.72–7.64 (m, 2H), 7.61 (d, *J* = 8.7 Hz, 1H), 7.52 (t, *J* = 5.8 Hz, 1H), 7.40 (d, *J* = 8.7 Hz, 1H), 7.32–7.13 (m, 10H), 6.96 (s, 3H), 4.90–4.73 (m, 2H), 4.54 (d, *J* = 5.1 Hz, 7H), 4.49 (t, *J* = 7.0 Hz, 1H), 4.39–4.33 (m, 3H), 3.79 (s, 9H), 3.24 (s, 1H), 3.20–3.16 (m, 2H), 3.11–3.07 (m, 1H), 3.05–2.88 (m, 5H), 2.82 (t, *J* = 12.3 Hz, 1H), 2.75–2.64 (m, 1H), 2.59 (d, *J* = 9.3 Hz, 1H), 2.35–2.33 (m, 1H), 2.19 (t, *J* = 7.3 Hz, 2H), 2.06–1.99 (m, 13H), 1.80 (t, *J* = 8.1 Hz, 6H), 1.73–1.59 (m, 2H), 1.53 (d, *J* = 12.5 Hz, 2H), 1.32–1.16 (m, 3H), 1.12–0.91 (m, 2H), 0.88–0.83 (m, 6H). Calculated ESI^+^ [M + 1H]^1+^: 1926.0, [M + 2H]^2+^: 963.5, [M + 3H]^3+^: 642.7. Measured ESI^+^ [M + 1H]^1+^: 1924.2, [M + 2H]^2+^: 962.7, [M + 3H]^3+^: 643.6. High-resolution full scan ESI^+^ MS spectra of the compound 11 and 12 as well as Product ion spectrum from the AIF are presented in the ESI (Fig. S9, S10, S12 and S13).†

### Radiochemistry

^68^Ga was eluted from an Eckert & Ziegler (E&Z Radiopharma GmbH) ^68^Ge/^68^Ga generator producing 200–400 MBq of [^68^Ga]GaCl_3_, using hydrochloric acid 0.1 M and collected in five different fractions of 1 mL. Capintec CRC-25R was used as radiation counter. Radioactive HPLC was performed on an Agilent Technologies 1260 Infinity system equipped with degasser, UV detector (220 nm) and a radioactive detection (Bioscan Inc. B-FC-3200 photomultiplier tube detector). Laura software (LabLogic Systems Ltd) was used to collect and analyze all Radio-HPLC data. The reverse-phase method used is shown in Table S1 (ESI).† A Luna® C_18_(2) 100 Å LC column with 5 μm particle size and column dimensions of 100 × 2 mm obtained from Phenomenix UK Ltd was used for the analytical reverse-phase HPLC. Radio instant thin layer chromatography (ITLC) was developed on Agilent Technologies glass microfibre chromatography paper impregnated with silica gel and analyzed using a Lablogic Flow-count TLC scanner and a BioScan B-FC-3200 PMT detector and Laura software. Two different ITLC methods were used: (1) the acetate method (1 M ammonium acetate in water/methanol 1 : 1); (2) citrate method (0.175 M citric acid, 0.325 M trisodium citrate in water).

### Integrin αvβ6-peptide-THP (11 and 12) radiolabeling

11 and 12 were dissolved in water producing a stock solution of 1 μg μL^−1^. To 20 μL of stock solution (*i.e.* 20 μg of 11 or 12) was added 200 μL of [^68^Ga]GaCl_3_ (0.1 M HCl; corresponding to ∼30 MBq ^68^Ga, to give a specific activity of ∼3 GBq μmol^−1^) and 40 μL of 2.0 M ammonium acetate (99.999% trace metals basis) the pH was checked to ensure it was in the range 6.5–7.5. The mixture was agitated and left at room temperature, before final analysis with HPLC-C_18_ and ITLC at 5 min and 35 min.

### Cell binding experiments

BxPC-3 were plated in 12-well plates at a density of 5 × 10^5^ cells per well in 1 mL of complete culture medium, cultured overnight at 37 °C and 5% CO_2_. Radiolabelling with ^68^Ga was done as described above, and a solution containing 15 MBq in 1.0 mL was prepared by dilution with distilled H_2_O. Eleven inhibitor (unlabelled peptide) concentrations were tested to estimate the IC_50_ value at 0.1, 0.5, 1, 5, 10, 50, 100, 250, 500, 1000, and 5000 nM. Four wells were used for each concentration (*n* = 4), prepared in 500 μL of complete media. Following the addition of the inhibitor, 20 μL of the radiolabelled peptide solution was added to each well, corresponding to 300 kbq per well (33 nM of the labelled peptide per well). The cells were then incubated for an hour at the same above-mentioned conditions. Following incubation, media were removed from the wells, and the cells were washed twice with cold PBS (500 μL per washing). For each well, the media and the washings were collected together. After the second wash, 500 μL of 1 M NaOH solution was added to each well to lyse the cells, and cell lysates were retrieved 10 minutes later (15k g min^−1^). A final wash with PBS (500 μL per well) was undertaken and collected in the same tube as the cell lysate. All tubes (containing either medium and washings or lysate and washings) were gamma-counted using a Wallac 1282 Compugamma Universal Gamma Counter. The resulting values were analyzed using Graphpad Prism 9 software, and a non-linear regression curve was generated, from which the IC_50_ values (log IC_50_: −6.72 ± 0.20 for 11 and −6.58 ± 0.29 for 12) were obtained.

## Conflicts of interest

There are no conflicts to declare.

## Supplementary Material

DT-051-D2DT00980C-s001
