# Supplementary material for: Targeting integrin αvβ6 with gallium-68 tris (hydroxypyridinone) based PET probes
Source: Dalton Trans. 2022 Aug 11;51(34):12796–803. doi: 10.1039/d2dt00980c (PMC9425781; doi:10.1039/d2dt00980c)
Supplement: DT-051-D2DT00980C-s001 [file DT-051-D2DT00980C-s001.pdf]

Electronic Supplementary Material (ESI) for Dalton Transactions.
This journal is © The Royal Society of Chemistry 2022

Targeting Integrin αvβ6 with Gallium-68 tris(hydroxypyridinone) based PET Probes

Giuseppe Floresta,[a,b] Siham Memdouh,[a] Truc Pham,[c] Michelle T. Ma,[c] Philip J. Blower,[c] Robert C. Hider,[a] Vincenzo Abbate,[a] Agostino Cilibrizzi[a,d*]

[a]King's College London, Institute of Pharmaceutical Science, Franklin Wilkins Building, London SE1 9NH, United Kingdom
[b]Department of Drug and Health Sciences, University of Catania, Catania, Italy
[c]King's College London, Division of Imaging Sciences and Biomedical Engineering, Fourth Floor Lambeth Wing, St Thomas' Hospital, London SE1 7EH, United Kingdom
[d]Centre for Therapeutic Innovation, University of Bath, Bath, UK

Table of contents

Figure S1. iTLCs chromatograms for molecule **12** 2

Figure S2. radioHPLC chromatogram for molecule **12** 2

Figure S3. radioHPLC chromatogram for molecule **11** 3

Table S1. Method 1 for radio HPLC 3

Figure S4. Structure, HPLC chromatogram (281 nm) and mass spectrum (ESI$^+$)of molecule **7** 4

Figure S5. ^{1}H NMR of molecule **7** 5

Figure S6. Mass spectrum (ESI+) and structure of **9** 6

Figure S7. Structure, HPLC chromatogram (PDA) and mass spectrum (ESI$^+$)of molecule **10** 7

Figure S8. Structure, HPLC chromatogram (281 nm) and mass spectrum (ESI$^+$)of molecule **11** 8

Figure S9. High-resolution full scan ESI+ MS spectra of the compound 11 and a zoom view of the isotopic pattern of the precursor product 9

Figure S10. Product ion spectrum (11) from the AIF at collision energy 30 10

Figure S11. Structure, HPLC chromatogram (PDA) and mass spectrum (ESI$^+$)of molecule **12** 11

Figure S12. High-resolution full scan ESI+ MS spectra of the compound 12 and a zoom view of the isotopic pattern of the precursor product 12

Figure S13. Product ion spectrum (12) from the AIF at collision energy 30 13

Figure S14. ^{1}H NMR of molecule **11** 14

Figure S15. ^{1}H-^{1}H COSY NMR of molecule **11** 14

Figure S16. ^{1}H-^{13}C HSQC NMR of molecule **11** 15

Figure S17. ^{1}H NMR of molecule **12** 16

Figure S18. ^{1}H-^{1}H COSY NMR of molecule **12** 16

Figure S19. ^{1}H-^{13}C HSQC NMR of molecule **12** 17

Tables S1-S2. Nonlinear regression results and statistics for molecules **11** and **12** 18

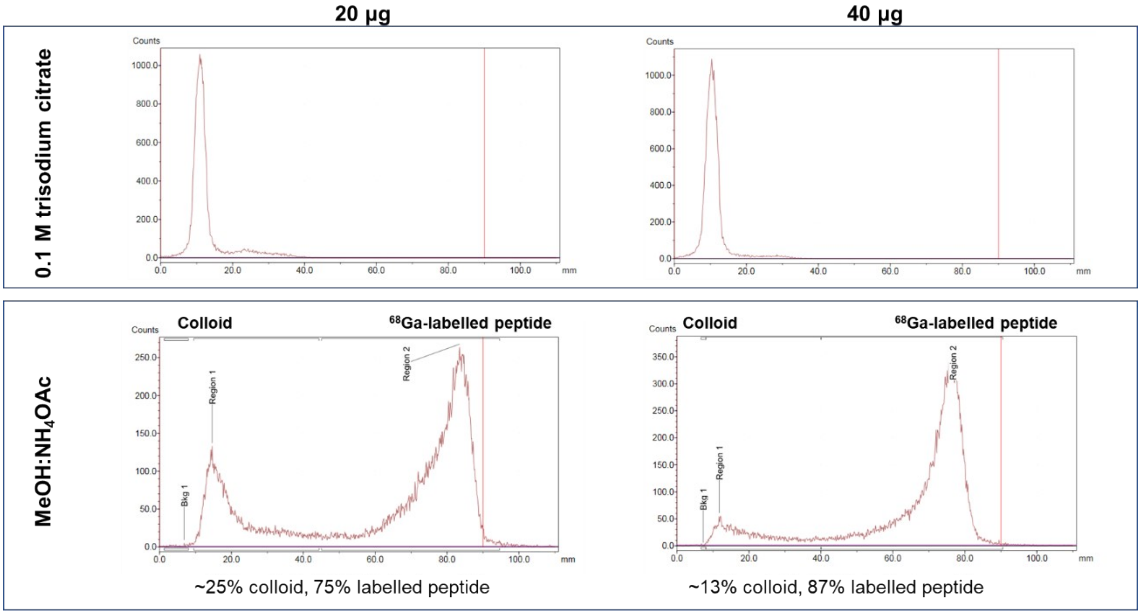

Figure S1. iTLCs chromatograms for molecule **12**.

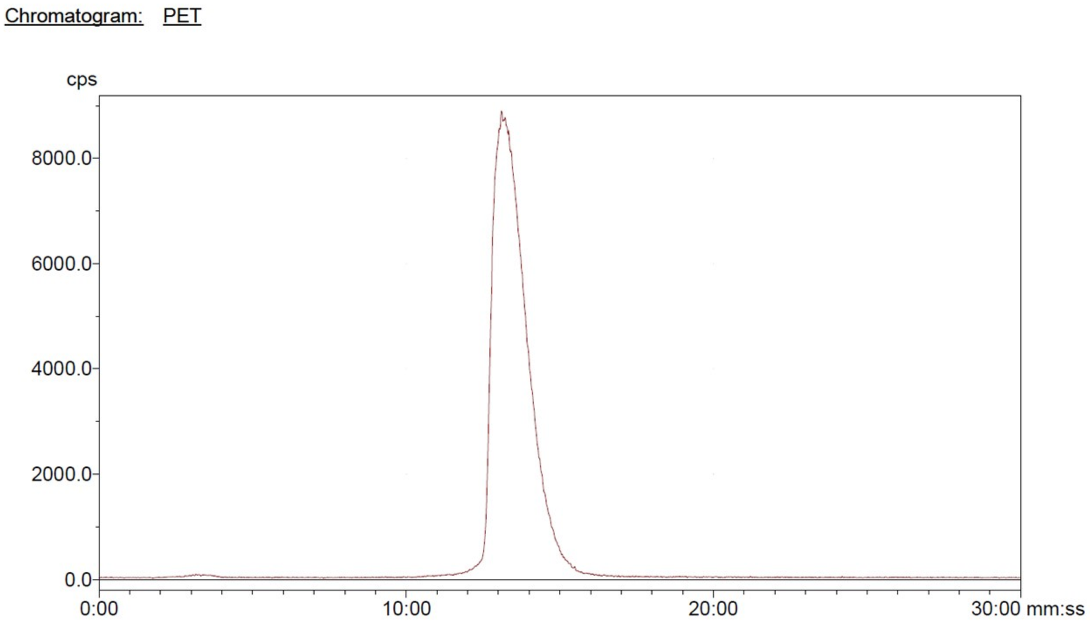

Figure S2. radioHPLC chromatogram for molecule **12**.

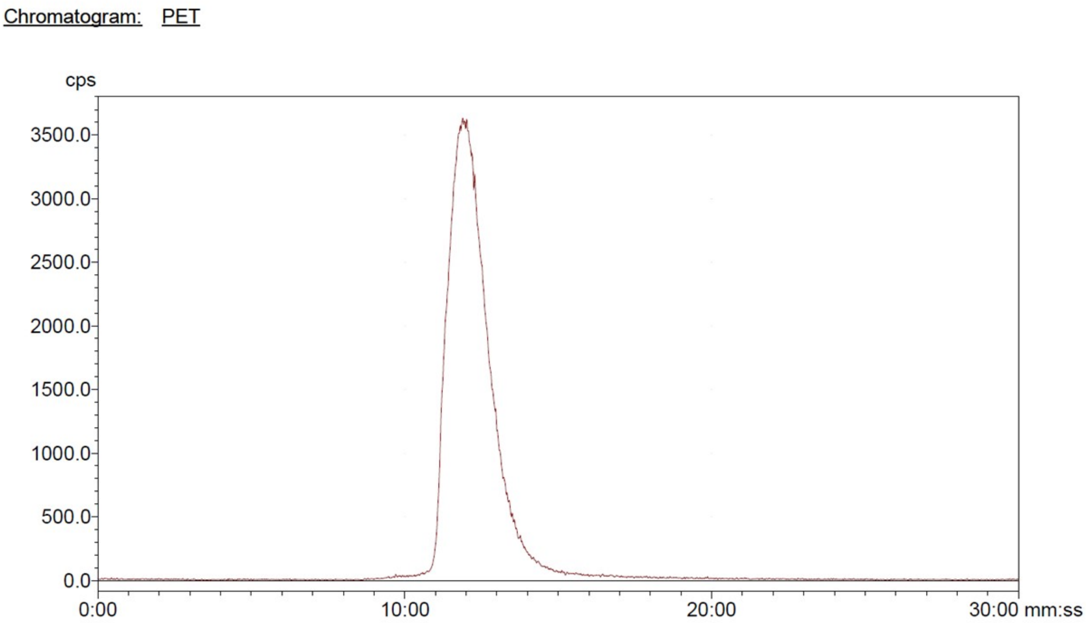

Figure S3. radioHPLC chromatogram for molecule **11**.

Table S1. Method 1 for radio HPLC: A = water + 0.1% trifluoroacetic acid. B = acetonitrile + 0.1% trifluoroacetic acid. Flow rate: 0.3 mL/min.

**Time / min**	**Solvent %**	
	**A**	**B**
0	100	0
20	10	90
23	10	90
25	100	0
30	100	0

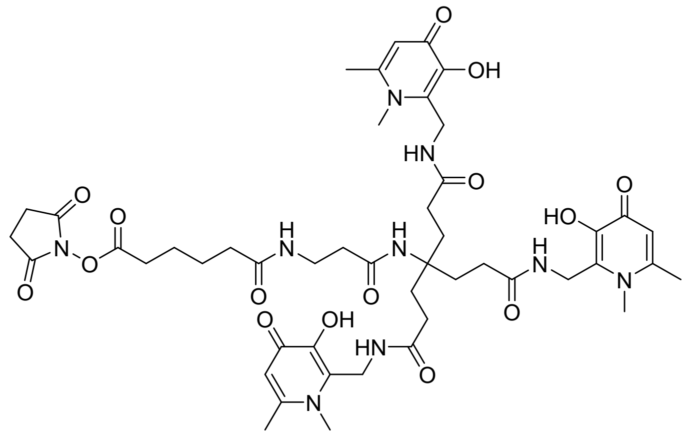

Chemical Formula: $C_{47}H_{63}N_9O_{15}$
Exact Mass: 993.4444

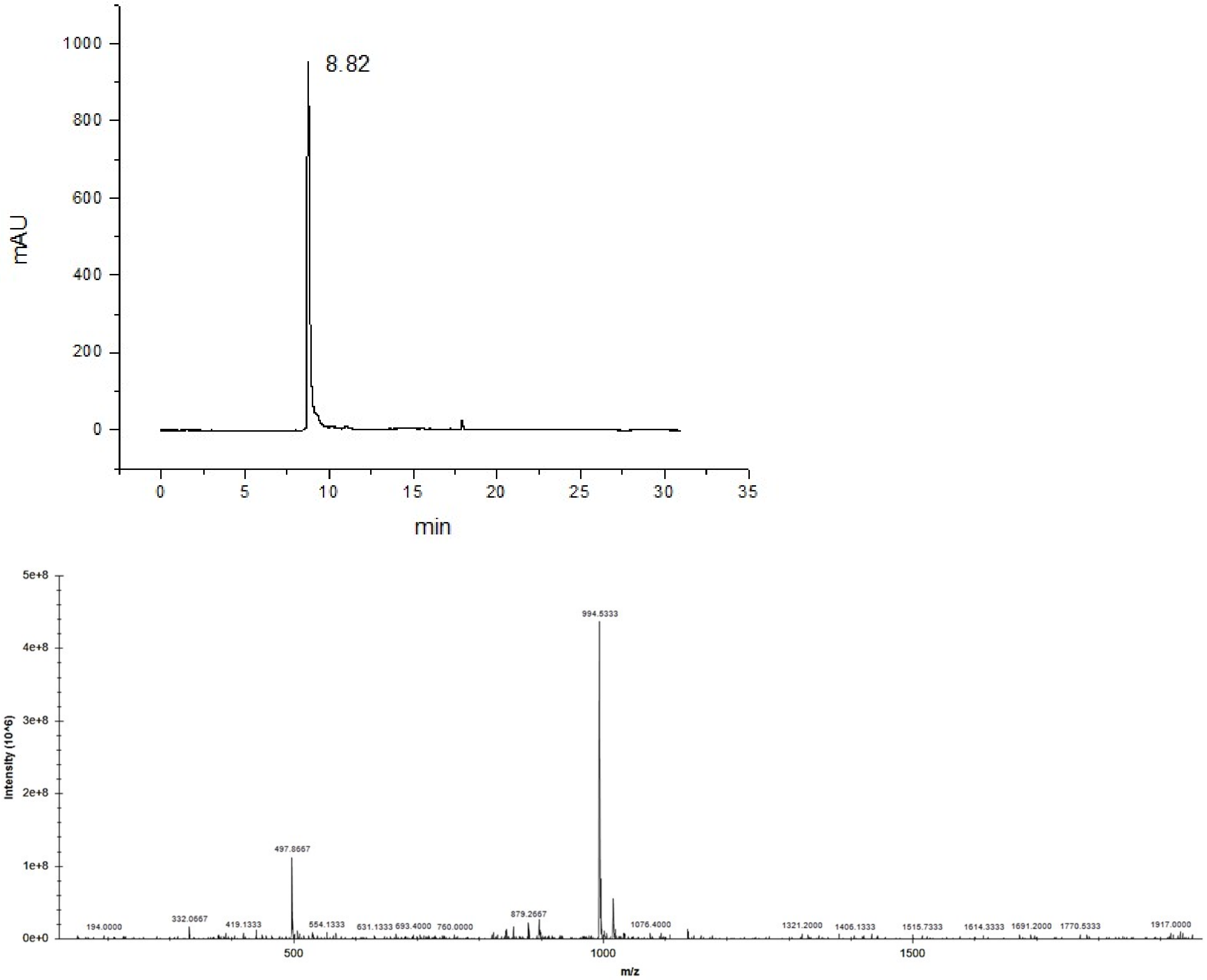

Figure S4. Structure, HPLC chromatogram (281 nm) and mass spectrum (ESI$^+$)of molecule **7**.

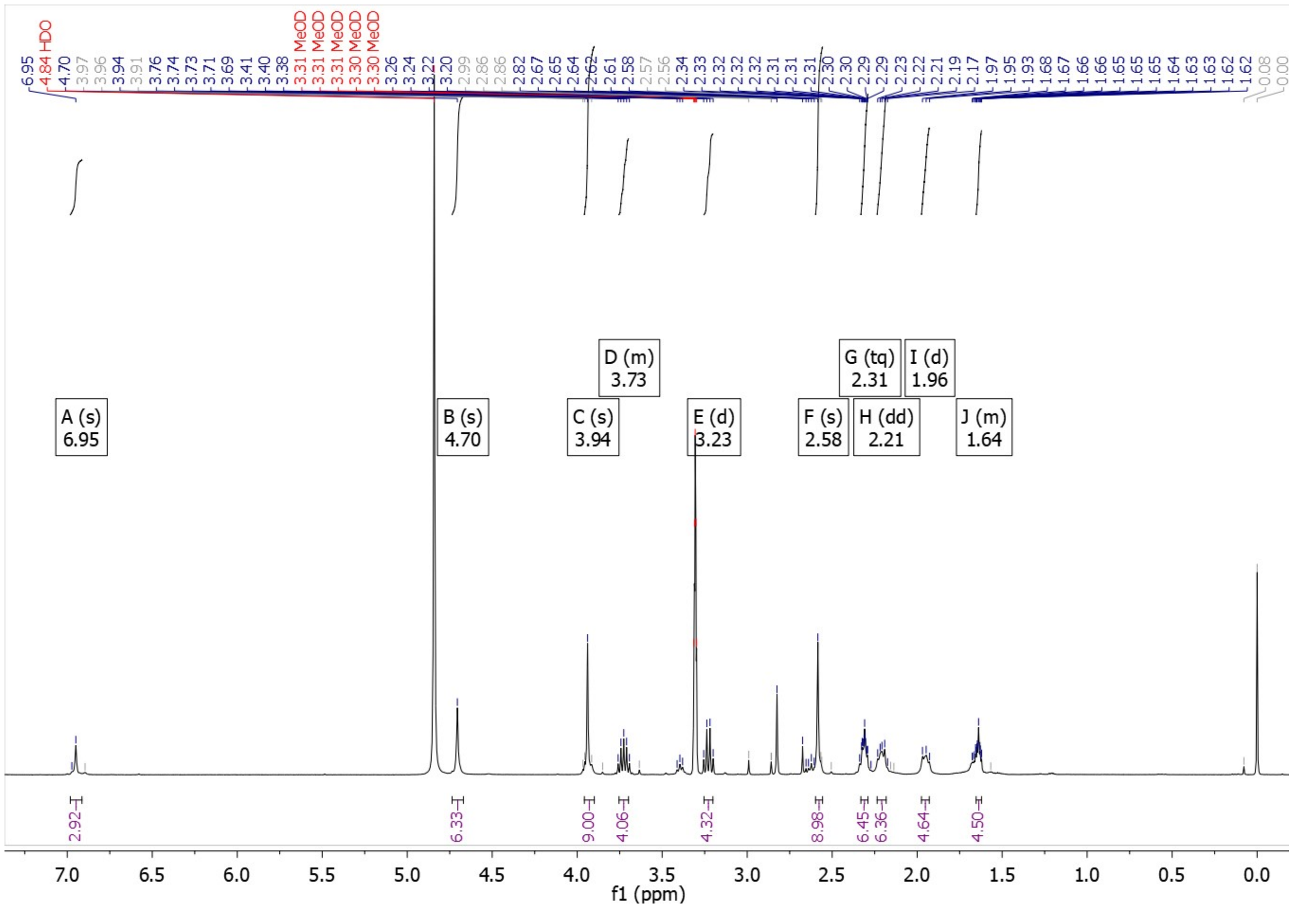

Figure S5. ^{1}H NMR of molecule **7**.

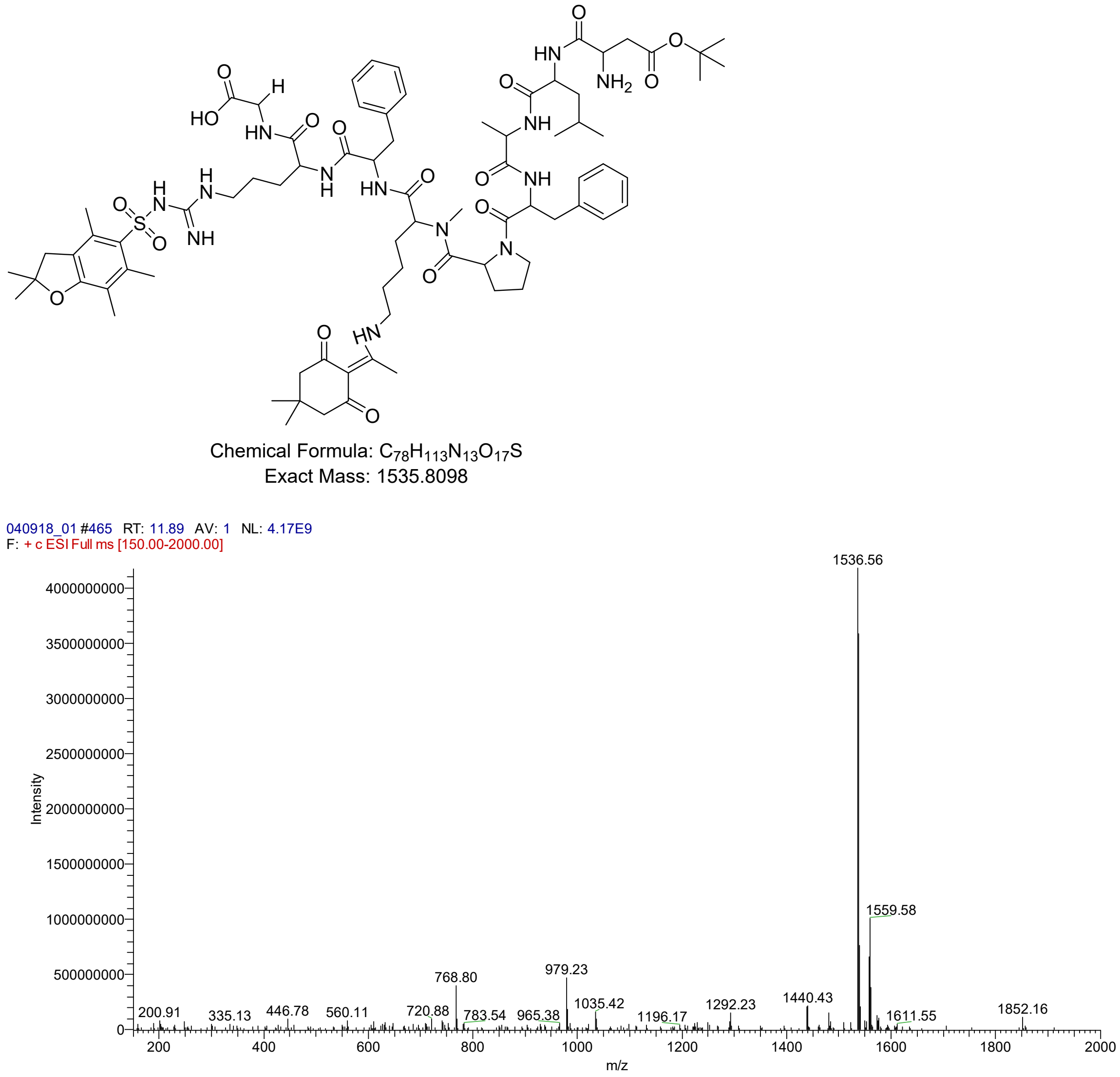

Figure S6. Mass spectrum (ESI+) and structure of **9**.

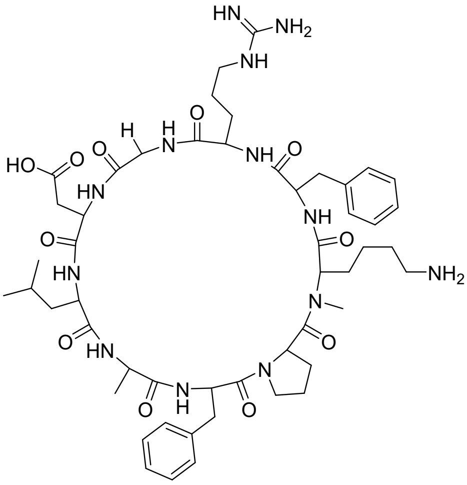

Chemical Formula: $C_{51}H_{75}N_{13}O_{11}$
Exact Mass: 1045.5709

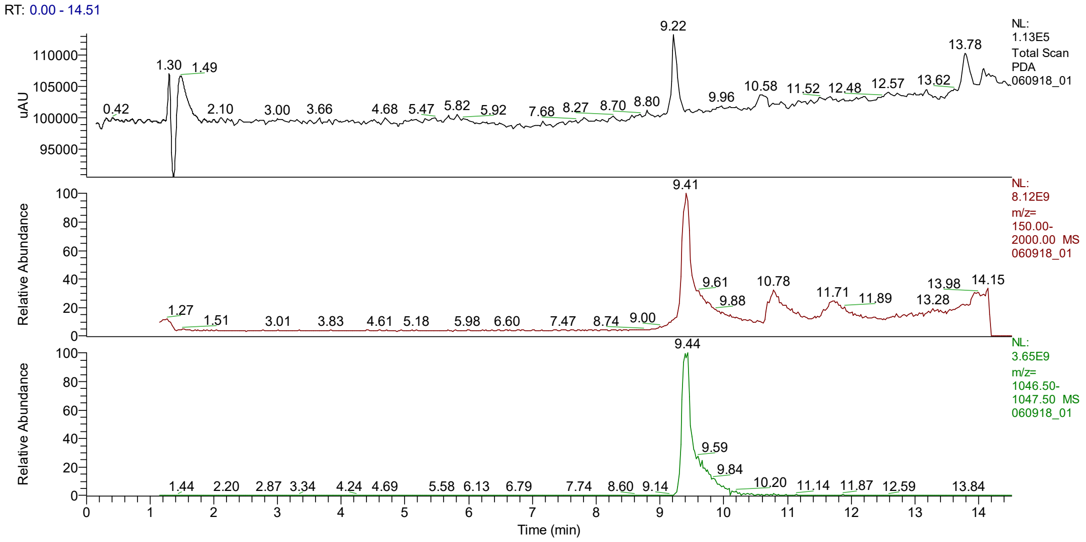

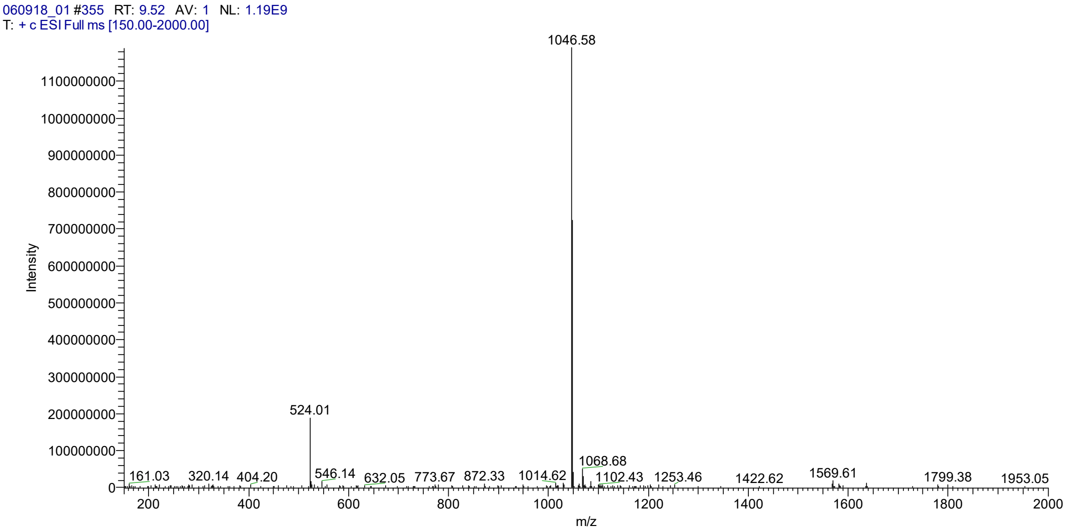

Figure S7. Structure, HPLC chromatogram (PDA) and mass spectrum (ESI^+)of molecule **10**.

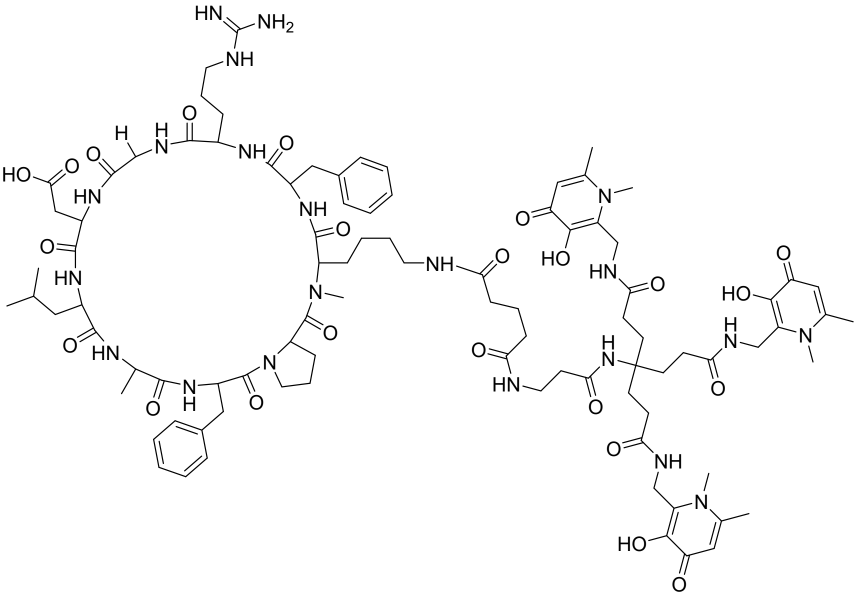

Chemical Formula: $C_{93}H_{131}N_{21}O_{23}$
Exact Mass: 1909.9727

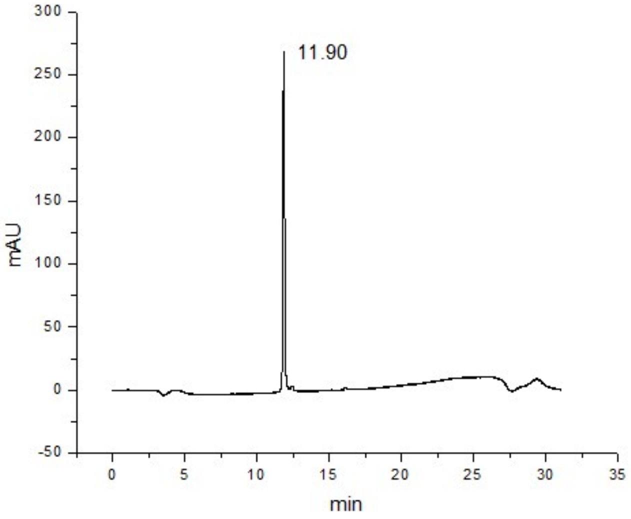

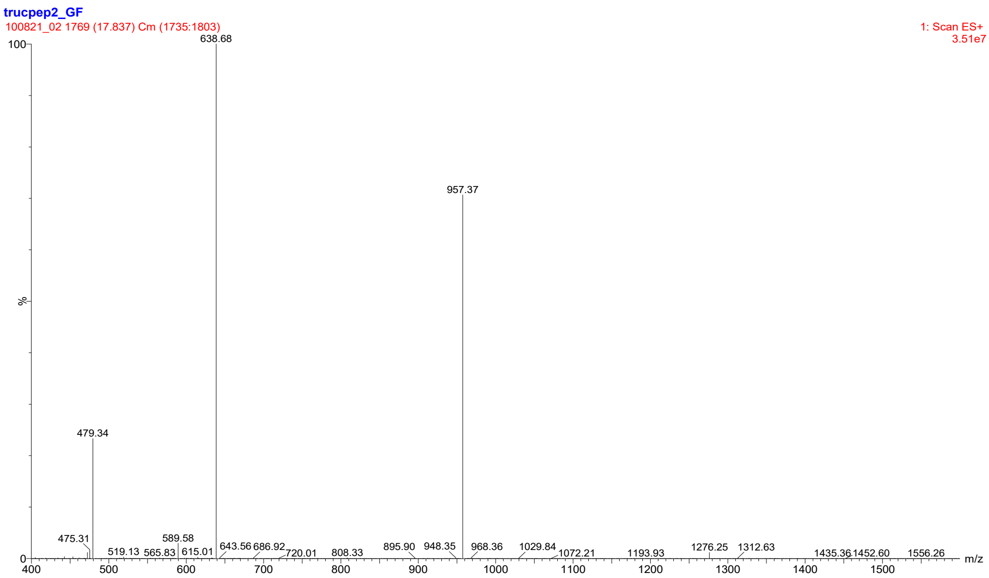

Figure S8. Structure, HPLC chromatogram (281 nm) and mass spectrum (ESI$^+$) of molecule **11**.

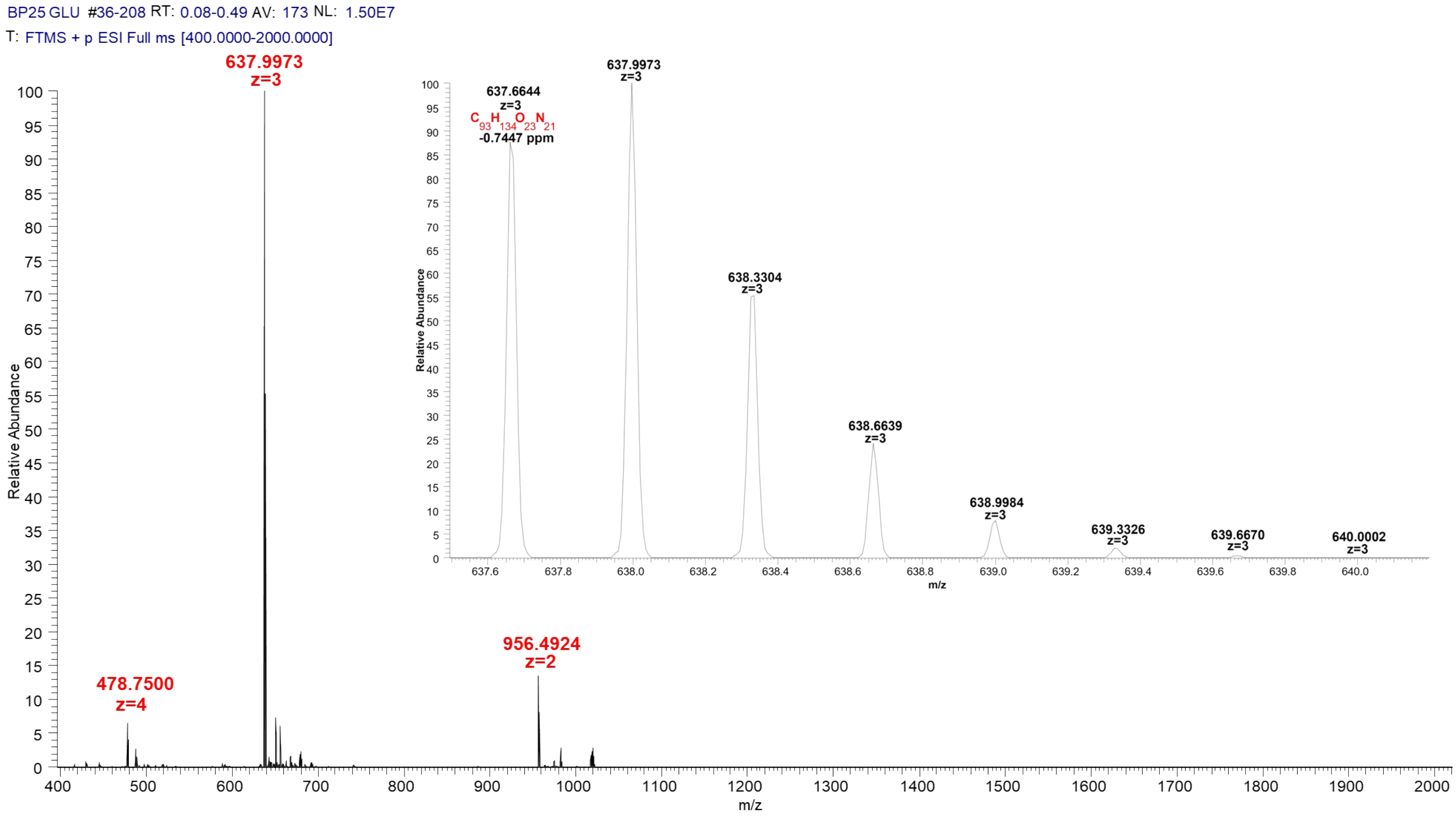

Figure S9. High-resolution full scan ESI+ MS spectra of the compound 11 and a zoom view of the isotopic pattern of the precursor product

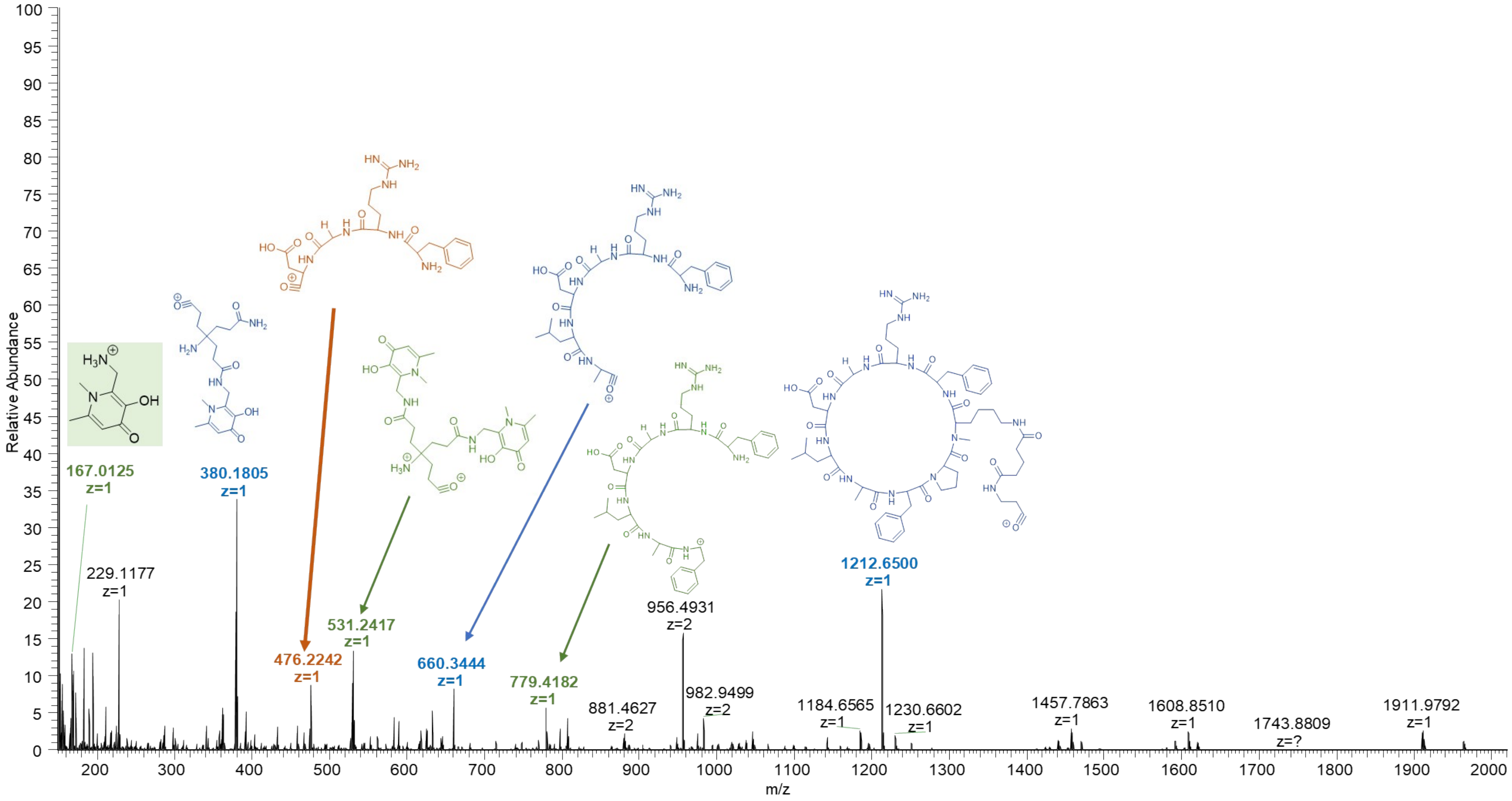

Figure S10. Product ion spectrum from the AIF at collision energy 30

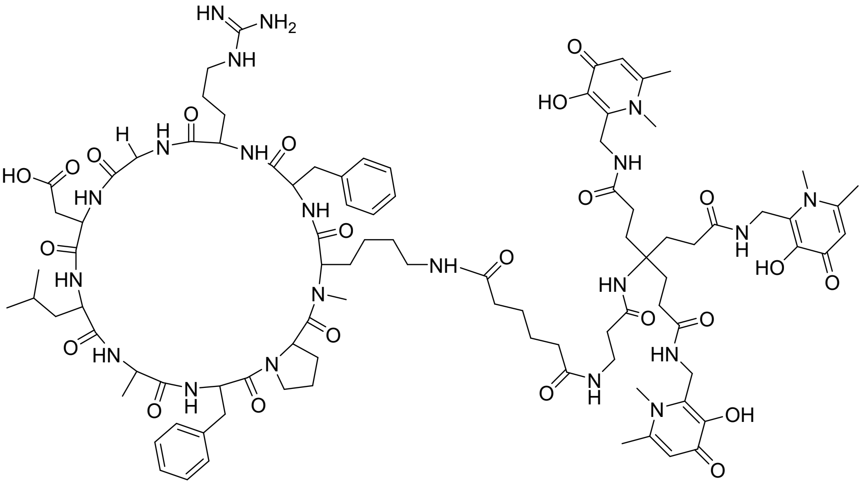

Chemical Formula: $C_{94}H_{133}N_{21}O_{23}$
Exact Mass: 1923.9883

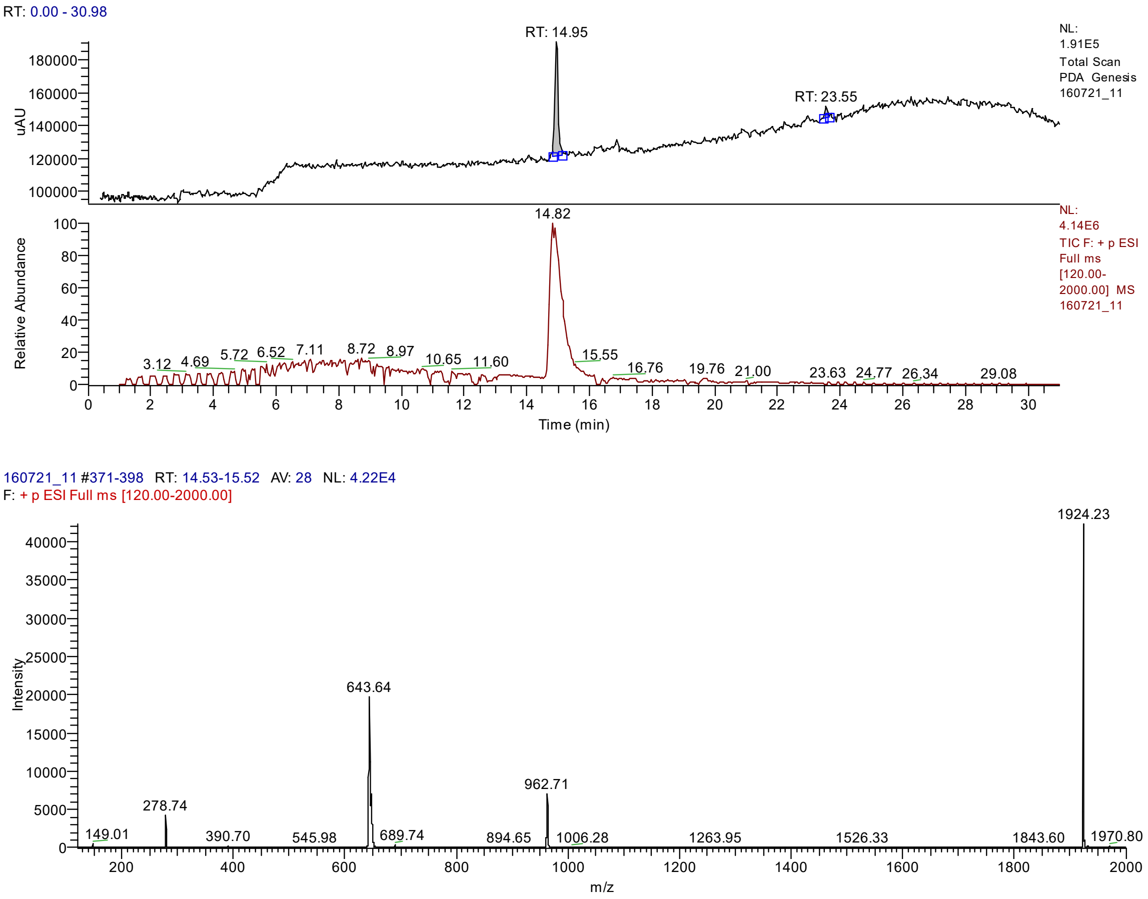

Figure S11. Structure, HPLC chromatogram (281 nm) and mass spectrum (ESI$^+$) of molecule **12.**

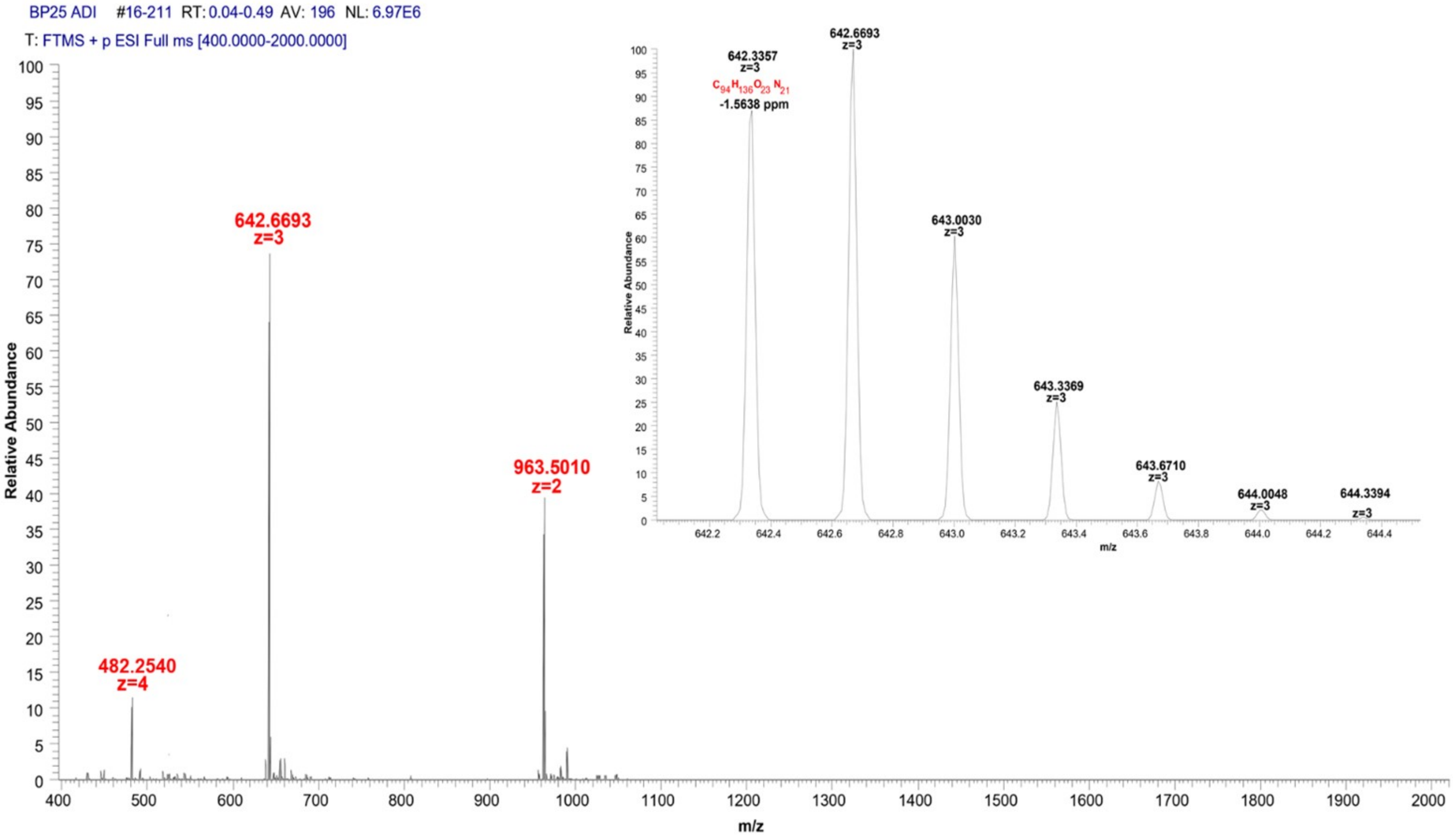

Figure S12. High-resolution full scan ESI+ MS spectra of the compound 12 and a zoom view of the isotopic pattern of the precursor product

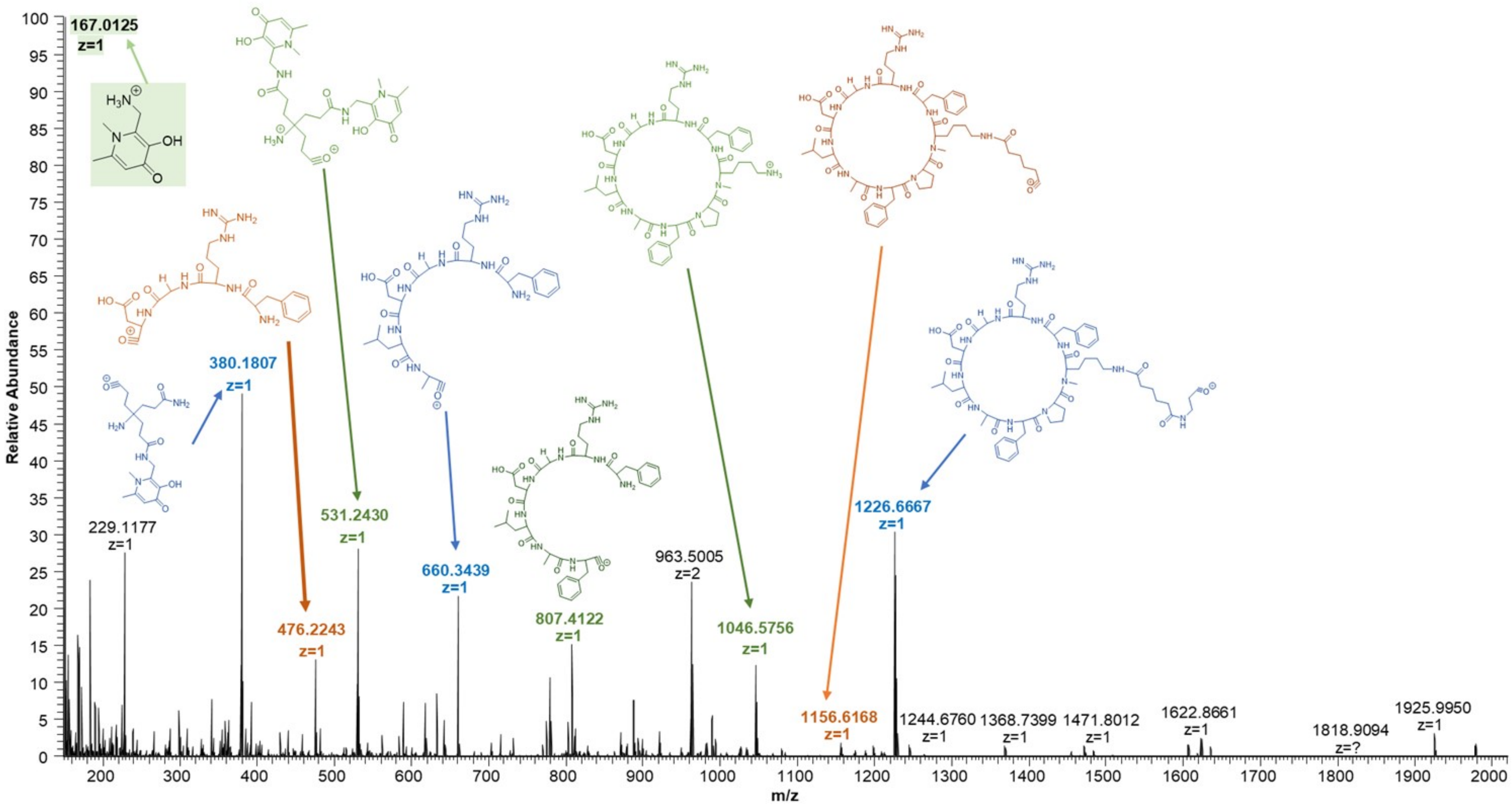

Figure S13. Product ion spectrum from the AIF at collision energy 30

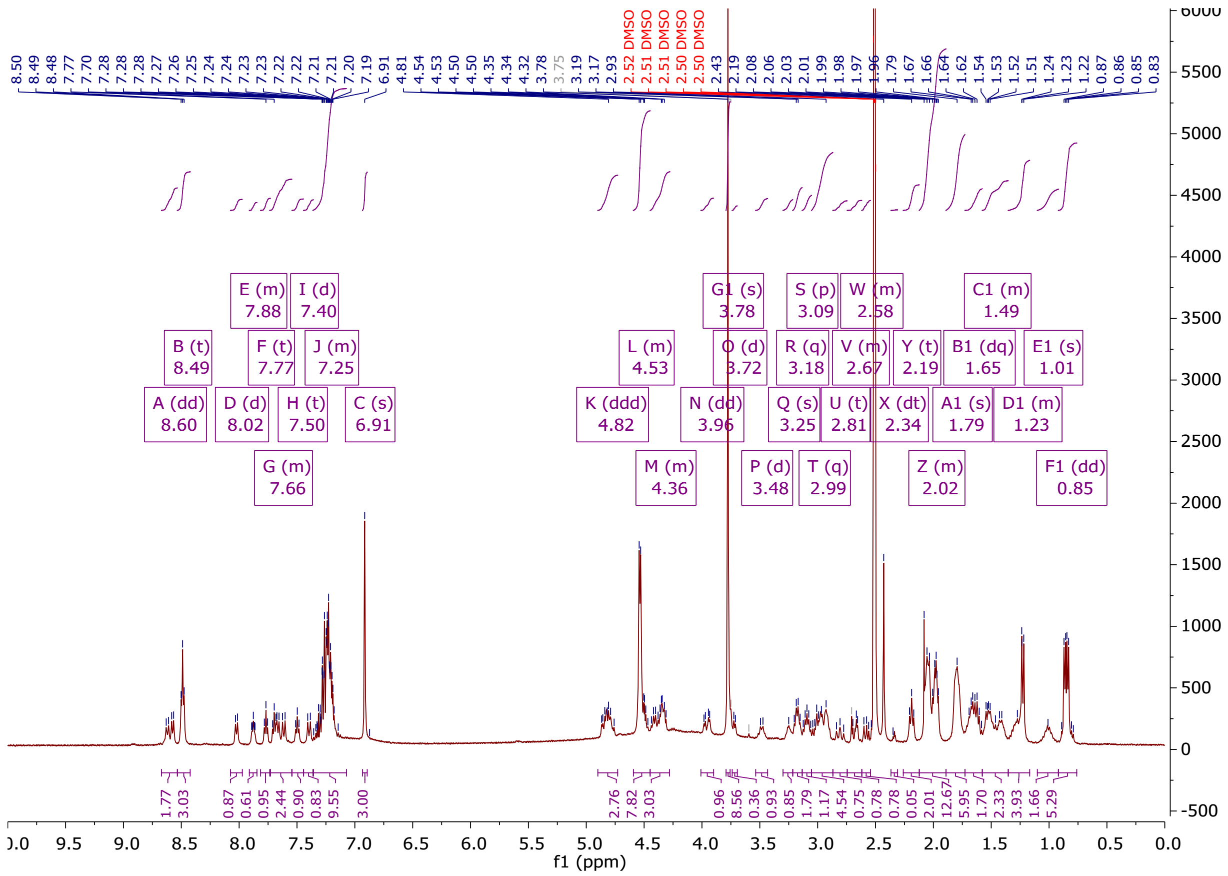

Figure S14. ^{1}H NMR of molecule **11.**

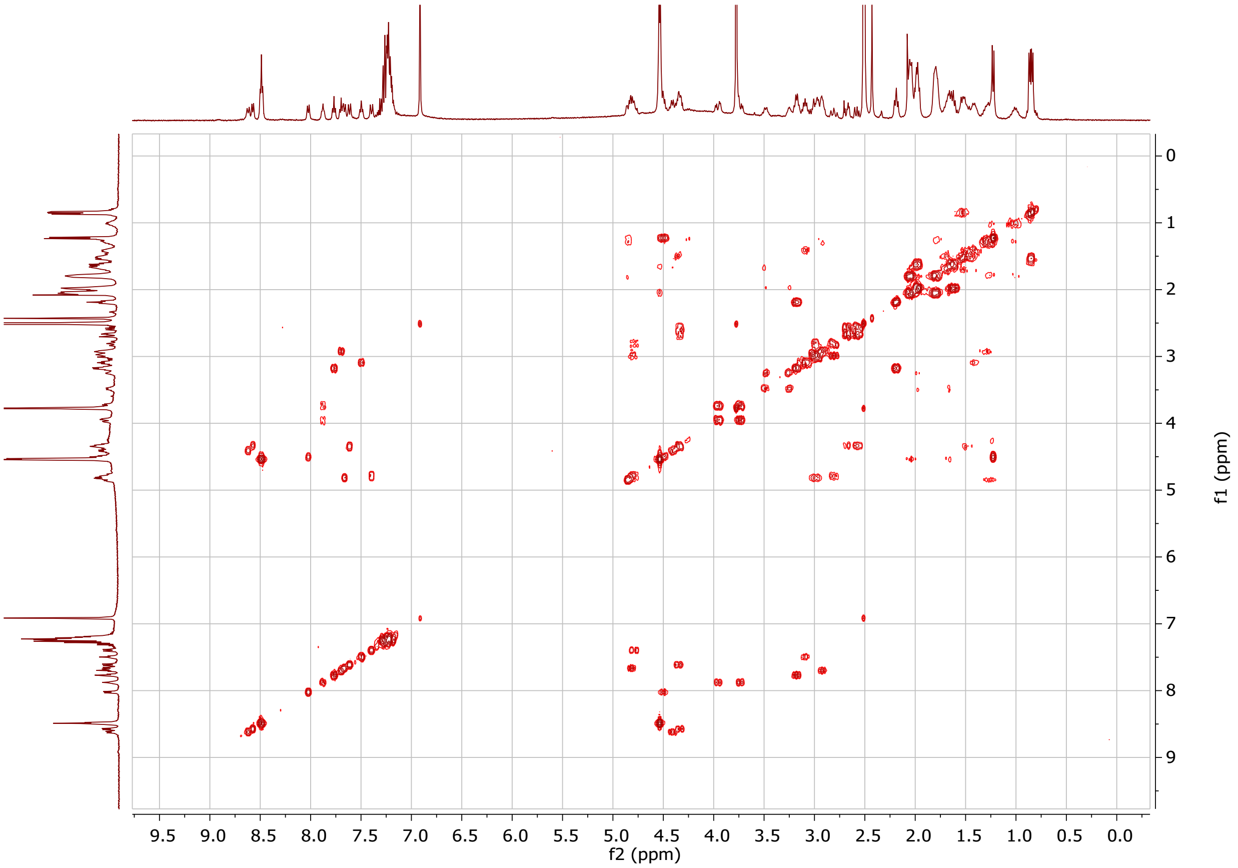

Figure S15. ^{1}H-^{1}H COSY NMR of molecule **11.**

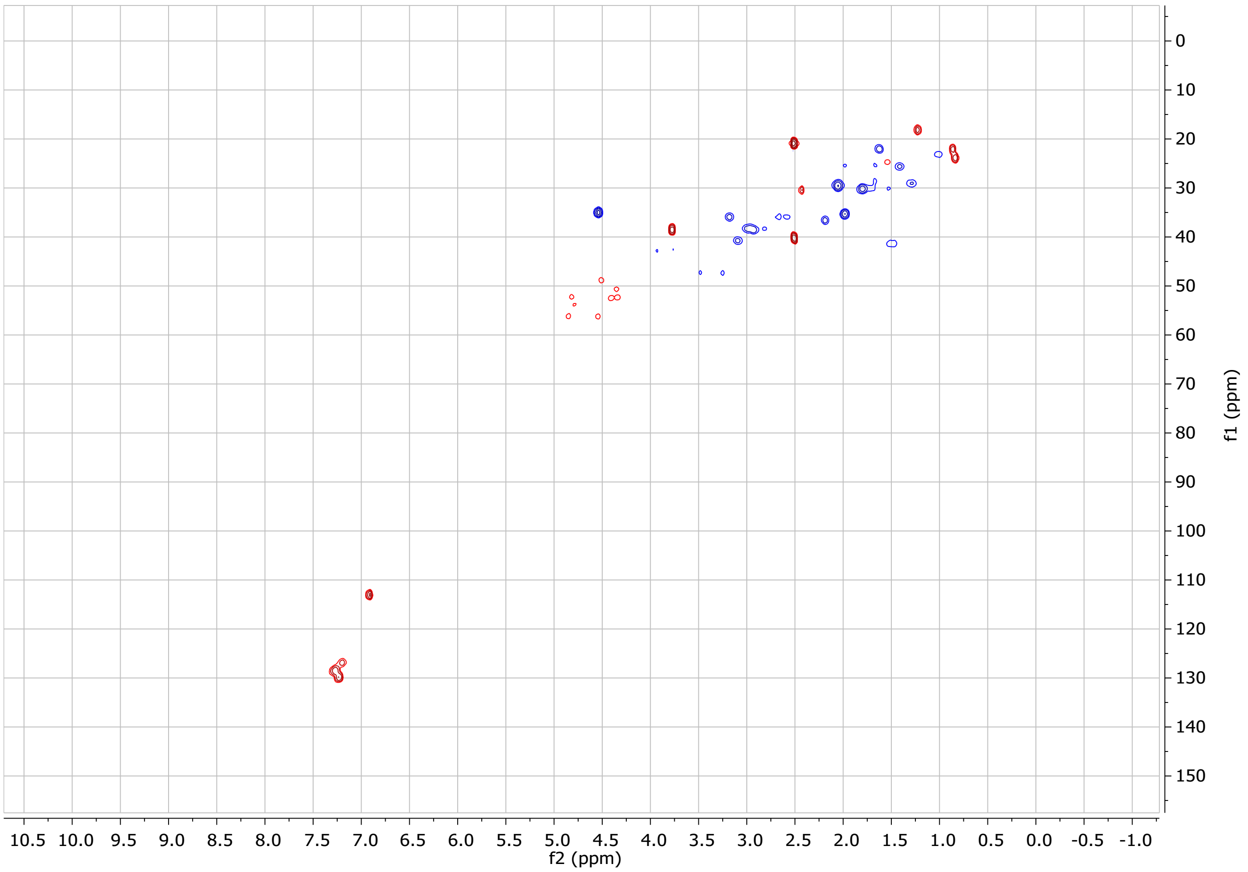

Figure S16. ^{1}H-^{13}C HSQC NMR of molecule **11.**

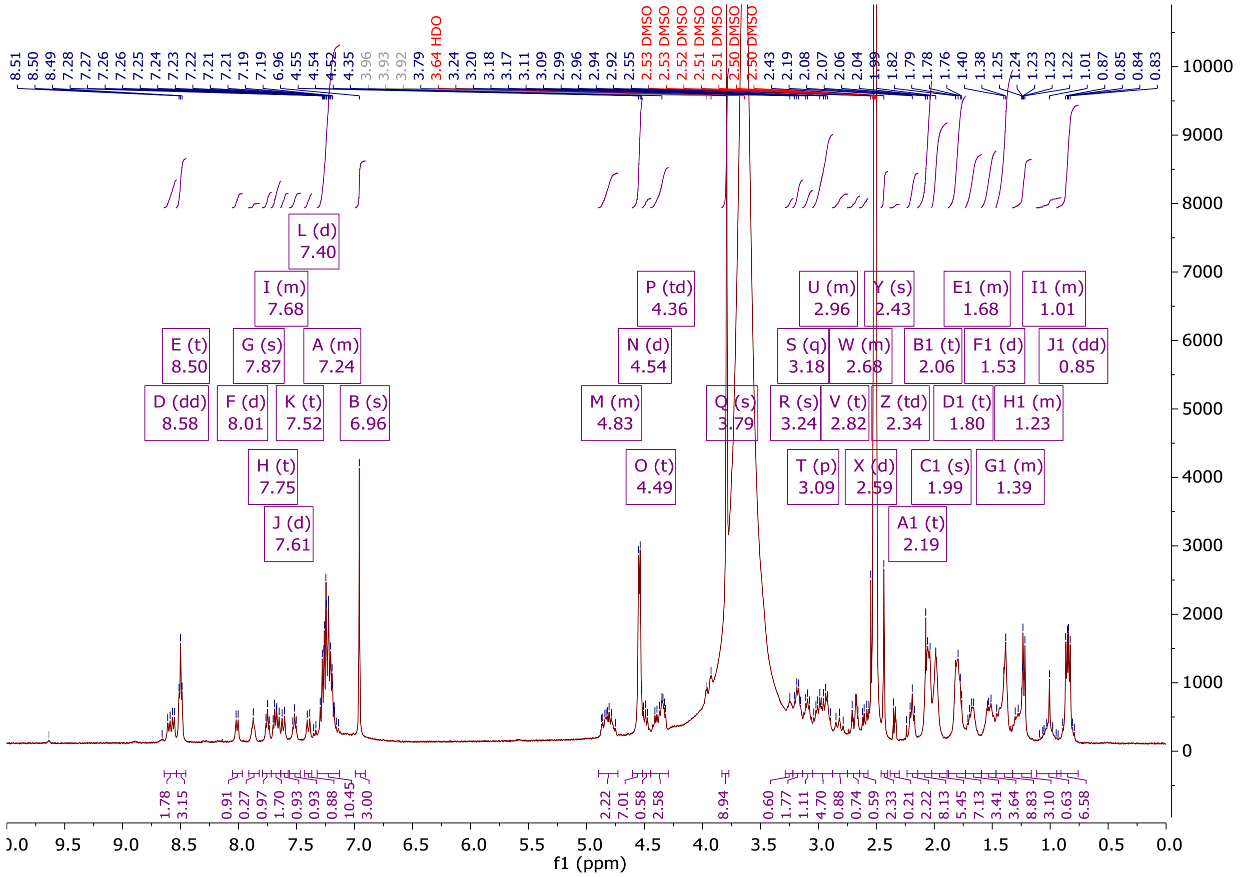

Figure S17. ^{1}H NMR of molecule **12.**

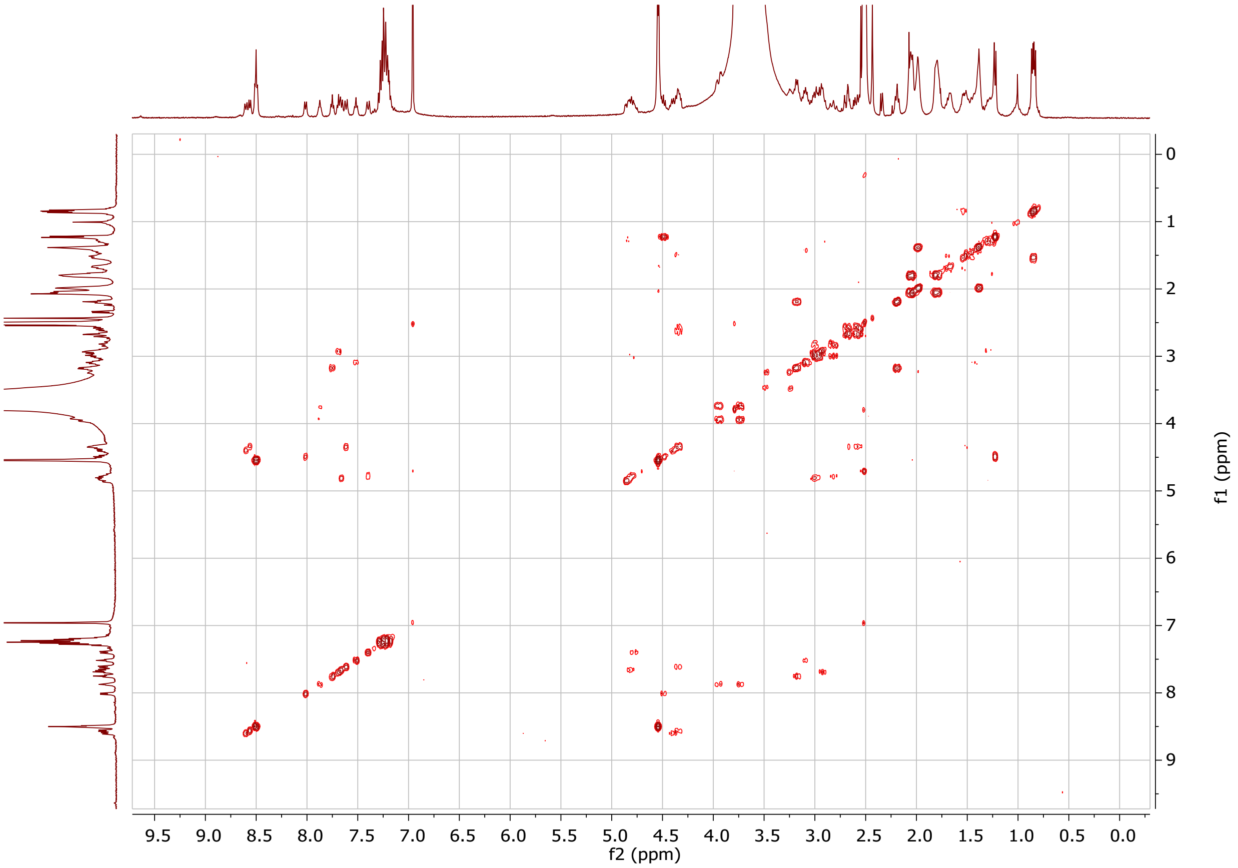

Figure S18. ^{1}H-^{1}H COSY NMR of molecule **12**

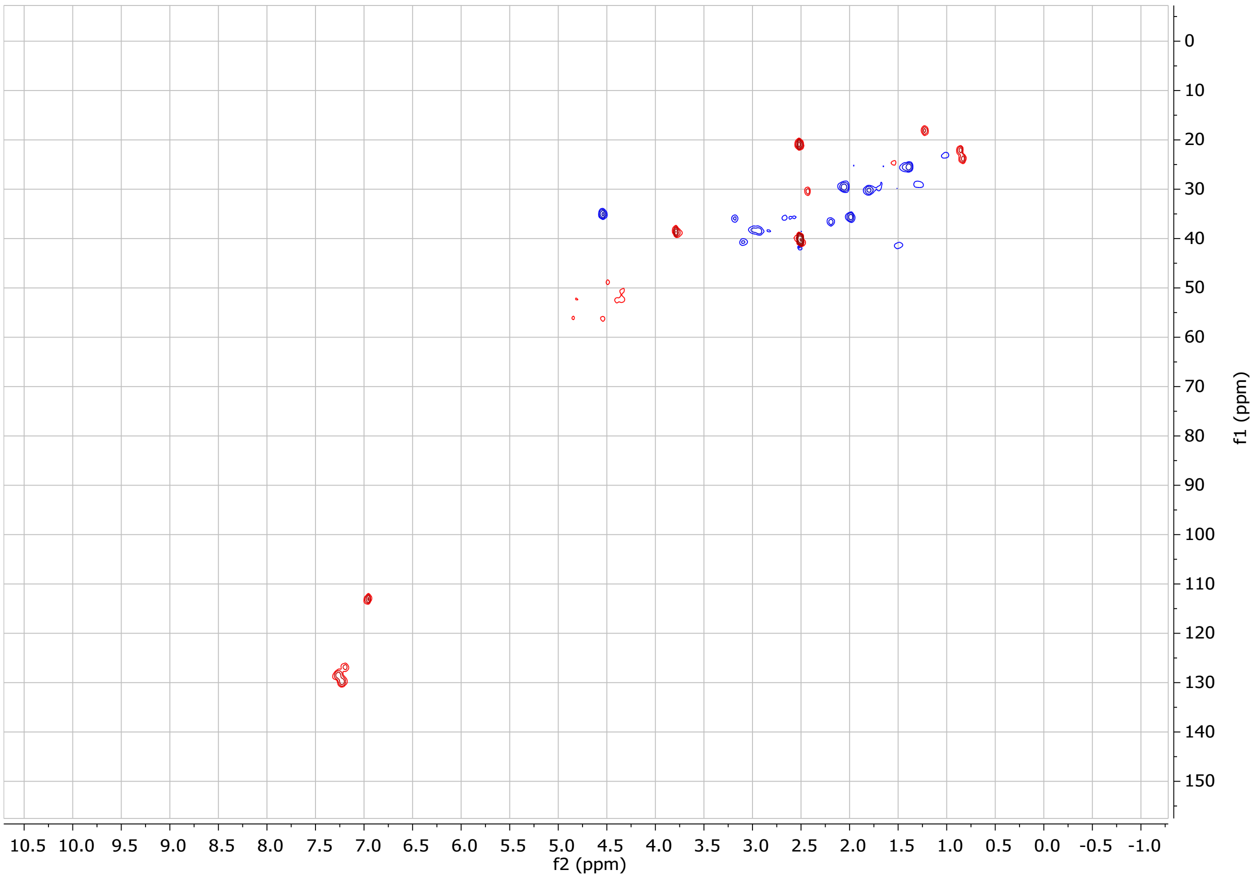

Figure S19. ^{1}H-^{13}C HSQC NMR of molecule **12**

Table S1. Nonlinear regression results and statistics for molecule **11**

**Best-fit values**	
Bottom	0.06221
Top	0.1267
LogIC50	-6.717
HillSlope	-1.239
IC50	1.917e-007
Span	0.06445
**Std. Error**	
Bottom	0.01017
Top	0.004453
LogIC50	0.1982
HillSlope	0.5820
Span	0.01175
**95% CI (asymptotic)**	
Bottom	0.04141 to 0.08300
Top	0.1175 to 0.1358
LogIC50	-7.123 to -6.312
HillSlope	-2.429 to -0.04897
IC50	7.541e-008 to 4.875e-007
Span	0.04041 to 0.08849
**Goodness of Fit**	
Degrees of Freedom	29
R squared	0.7233
Sum of Squares	0.007329
Sy.x	0.01590

Table S2. Nonlinear regression results and statistics for molecule **12**

**Best-fit values**	
Bottom	0.1165
Top	0.3176
LogIC50	-6.580
HillSlope	-0.8516
IC50	2.630e-007
Span	0.2012
**Std. Error**	
Bottom	0.04302
Top	0.01334
LogIC50	0.2904
HillSlope	0.3994
Span	0.04901
**95% CI (asymptotic)**	
Bottom	0.02951 to 0.2034
Top	0.2906 to 0.3446
LogIC50	-7.167 to -5.993
HillSlope	-1.659 to -0.04442
IC50	6.808e-008 to 1.016e-006
Span	0.1021 to 0.3002
**Goodness of Fit**	
Degrees of Freedom	40
R squared	0.6798
Sum of Squares	0.08743
Sy.x	0.04675
